# Population coding of valence in the basolateral amygdala

**DOI:** 10.1038/s41467-018-07679-9

**Published:** 2018-12-05

**Authors:** Xian Zhang, Bo Li

**Affiliations:** Cold Spring Harbor Laboratory, Cold Spring Harbor, New York, NY 11724 USA

## Abstract

The basolateral amygdala (BLA) plays important roles in associative learning, by representing conditioned stimuli (CSs) and unconditioned stimuli (USs), and by forming associations between CSs and USs. However, how such associations are formed and updated remains unclear. Here we show that associative learning driven by reward and punishment profoundly alters BLA population responses, reducing noise correlations and transforming the representations of CSs to resemble the valence-specific representations of USs. This transformation is accompanied by the emergence of prevalent inhibitory CS and US responses, and by the plasticity of CS responses in individual BLA neurons. During reversal learning wherein the expected valences are reversed, BLA population CS representations are remapped onto ensembles representing the opposite valences and predict the switching in valence-specific behaviors. Our results reveal how signals predictive of opposing valences in the BLA evolve during learning, and how these signals are updated during reversal learning thereby guiding flexible behaviors.

## Abstract (web summary)

It is unclear how the basolateral amygdala (BLA) contributes to behaviors driven by aversive or appetitive stimuli. Here, authors simultaneously record the activities of ensembles of BLA neurons in behaving mice to show that distinct but spatially intermingled BLA populations respond to either reward or punishment and that associative learning transforms BLA population activities to represent specific valences

## Introduction

Many stimuli in the environment have valences. For example, food and water are attractive to animals and humans under metabolic demand, whereas harm and punishment are inherently aversive^1–5^. Other environmental stimuli, such as a random sound or visual cue may not be attractive or aversive by nature. However, animals and humans have the ability to assign a positive or negative valence to an otherwise neutral stimulus (also known as conditioned stimulus, or CS) on condition that the stimulus is frequently associated with the occurrence of a good or bad consequence (also known as unconditioned stimulus, or US), and furthermore to revoke or reassign the valence if the stimulus-consequence contingency has changed^3,5–7^. This process, the core of which is known as associative learning, is fundamental for successfully foraging in a world filled with rewards, dangers, and uncertainties, because it enables an organism to use, on the basis of past experiences, arbitrary environmental cues (CSs) to predict beneficial or detrimental outcomes (USs), and moreover to flexibly update the predictions in the face of changes in CS–US contingencies^3,5–8^.

How the brain assigns valences to CSs according to CS–US contingencies, and updates the valences when the contingencies change, has been a subject of intensive study. Substantial evidence indicates that neurons in the basolateral amygdala (BLA) have an important role in such associative learning^9–14^. The BLA receives sensory inputs of all modalities, which can serve as CSs^6,11,15–22^, as well as inputs carrying appetitive or aversive information that may serve as USs^22–34^. Notably, recent studies suggest that BLA neurons (or at least some of them) responsive to appetitive and aversive USs—which are identified and targeted on the basis of expression of the immediate early gene *c-fos*^22,35,36^ or projection targets^37–40^—are hard-wired in valence-specific circuits, as activation of these neurons optogenetically induces behavioral responses conforming to the valences of the actual USs, and moreover can substitute for the USs to drive appetitive or aversive conditioning.

An emerging theme is that during associative learning CSs acquire the ability to activate the hard-wired US circuits, which in turn drives valence-specific behavioral responses^41^. Consistent with this idea, CS-evoked spiking activity in individual BLA neurons increases with appetitive or aversive conditioning, correlates with learning and represents the valence of the US^23,24,33^. Notably, recent studies have mapped the valence-specific BLA neurons onto distinct output pathways^39,40^, providing a circuit mechanism of how BLA neurons contribute to behaviors motivated by positive and negative valences.

Nevertheless, these studies focus on BLA neuronal responses in well-trained animals and do not provide information about how these valence-specific responses develop during learning, as it is challenging to track the same neurons throughout learning using the in vivo single unit recording techniques. Furthermore, the responses of individual neurons in single trials are noisy and thus do not reliably predict behavior on a trial-by-trial basis^42^. A recent study used imaging methods to record the activities of ensembles of BLA neurons throughout fear conditioning, and showed that BLA population activities in single trials provide a robust account for learning-induced freezing behavior^43^. Although this study did not examine the roles of the BLA in learning driven by reward, the novel approaches employed by the study provide an opportunity to determine the relationship between BLA population activities and the establishment of valence-specific behaviors, such as, for example, how population CS responses in the BLA evolve in naïve animals during learning to represent both positive and negative valences, and how these representations are dynamically updated in “real time” in response to changes in CS-–US contingencies and thus influence ongoing behaviors.

To address these issues, in the current study, we monitored the activities of ensembles of BLA neurons—reported with the genetically encoded calcium indicator GCaMP6f^44^—by imaging through gradient-index (GRIN) lenses^34,45,46^ implanted in the BLA in mice performing a Pavlovian associative learning task, in which the mice learned to associate one CS with reward, and the other with punishment. We subsequently imaged the activities of these neurons in a reversal learning procedure in which the valences initially assigned to the CSs were reversed. Our results illustrate how signals predictive of opposing valences in the BLA develop from naïve state during learning, and how these signals are updated on a trial-by-trial basis during reversal learning in a manner that they can be used to guide appropriate and flexible behavioral responses.

## Results

### The innate responses of BLA neurons to CSs and USs of opposing valences

**Fig. 1:**
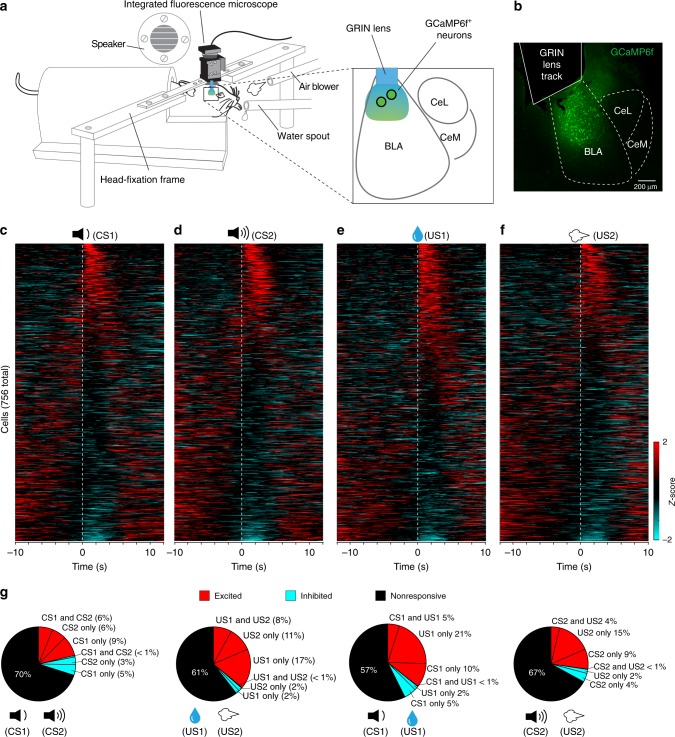
The innate responses of BLA neurons to CSs and USs. **a** A schematic of the setup for simultaneously monitoring behavioral and neuronal responses in head-restrained mice. We imaged GCaMP6 signals in BLA neurons through GRIN lenses in behaving mice using a miniature integrated fluorescence microscope mounted on the head. **b** A representative confocal image of a coronal brain section containing the BLA, in which the track of an implanted GRIN lens was on top of the BLA neurons expressing GCaMP6f. **c** Heatmaps of the activities (*z*-scores) for all neurons (*n* = 756 neurons, six mice) in trials in which CS1 was presented (indicated by the dashed line). Each row represents the temporal activities of one neuron. Neurons are sorted according to their average *z*-scores during the 1-s time window immediately after CS1 onset. **d**–**f** Same as (**c**), except that CS2 (**d**), US1 (water reward) (**e**), or US2 (air-puff) (**f**) was presented as the stimulus. **g** Pie charts showing the percent distributions of neurons responsive to different stimuli before learning. Note that at this stage, inhibitory responses were rare; and only a small percentage of neurons responded to both CS1 and US1, or to both CS2 and US2

To monitor neuronal activity in the BLA in behaving animals, we injected the BLA of wild type mice with an adeno-associated virus (AAV) expressing GCaMP6f (AAV1-Syn-GCaMP6f.WPRE.SV40) (Fig. 1a, b; Supplementary Fig. 1a). The GCaMP6f delivered by this virus was predominantly expressed in excitatory pyramidal neurons (only 4.9 ± 1% of GCaMP6f-expressing cells are GABAergic; *n* = 3 mice) (Supplementary Fig. 1b). We subsequently implanted a GRIN lens into the BLA and above the infected neurons (Fig. 1a, b; Supplementary Fig. 1a, c) in each of these mice. Four to six weeks after the surgery, we used a miniature integrated fluorescence microscope^45,46^ to record through the GRIN lenses dynamic GCaMP6f fluorescent signals from BLA neurons in these mice in wakefulness and under head-restraint (Methods). The constrained non-negative matrix factorization (CNMF) methods were used for imaging data processing before analysis, as previously described^34,47^ (Supplementary Video 1; Methods).

We first examined BLA neuronal activities in naïve mice in response to stimuli with different valences, including neutral tones (CS1s (2 kHz) and CS2s (10 kHz)), water rewards (US1s; note that mice were mildly water deprived) and aversive air-puffs blowing to the face (US2s) (Fig. 1a). BLA neurons exhibited diverse response profiles to these stimuli, with some responding to only one particular CS or US, while others responding to more than one stimuli (Supplementary Fig. 2). We classified a neuron as being responsive to a stimulus if the stimulus-evoked responses and the baseline activities, both represented as GCaMP6 signals (Δ*F*/*F*_0_), in this neuron were significantly different (*P* < 0.05, Wilcoxon signed-rank test) (see Methods). For further analyses, we used *z*-scores (calculated based on the mean and the standard deviation of the GCaMP6 signals in the entire recording duration; see Methods) to represent the dynamic activities in each neuron (Supplementary Fig. 3).

In naïve mice (*n* = 6), BLA neurons (*n* = 756) showed increased, decreased, or no change in activity in response to a particular stimulus (Fig. 1c–f; Supplementary Fig. 3). In particular, about 15% and 12% of neurons were excited by CS1 and CS2, respectively, 25% and 19% of neurons were excited by US1 and US2, respectively, while the majority showed only spontaneous activities and was classified as being “nonresponsive” (Fig. 1g). Only a small fraction of neurons was inhibited by any of these stimuli (<5%; Fig. 1g). There were also small fractions of neurons responsive to both CS1 and CS2 (6%), or to both US1 and US2 (8%) (Fig. 1g), and neurons responsive to both CS1 and US1 (5%), or to both CS2 and US2 (4%) (Fig. 1g).

It has been shown that different but partially overlapping populations of BLA neurons respond to reward and punishment^22,24,35,38^. Consistent with these studies, we found that among the US-responsive BLA neurons, 47.2% were excited by US1 (water reward) but not US2 (air-puff), and 30.6% were excited by US2 but not US1 (Supplementary Fig. 4a; Fig. 1g). There was also a smaller population (22.2%) excited by both USs (Supplementary Fig. 4a; Fig. 1g). This latter population may represent neurons responsive to salience^23^. Notably, the neurons excited by the USs of opposing valences were distributed in the field of view with no obvious anatomical separation (Supplementary Fig. 4a, b), a feature consistent with the findings in previous studies in which BLA neuronal activity was monitored with electrophysiological or imaging methods^24,43^, or based on the expression of the immediately early gene *c-fos* (ref. ^22^ but see ref. ^35^).

### Both reward learning and punishment learning link CS and US representations in BLA neurons

**Fig. 2:**
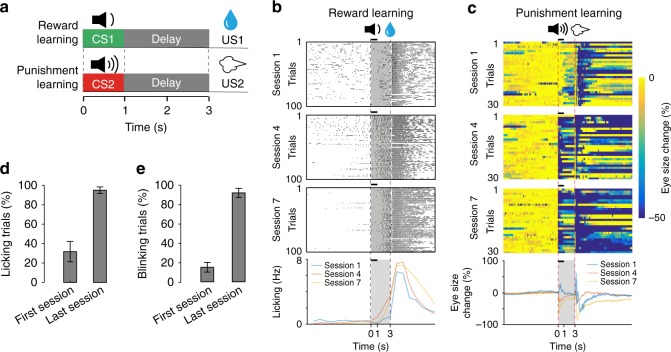
The behavioral task. **a** A schematic of the behavioral procedure, in which mice were trained to associate CS1 (a 2 kHz tone) with US1 (water reward), and CS2 (a 10 kHz tone) with US2 (air-puff blowing to the face). **b** Changes in licking behavior during the reward learning for a representative mouse. The upper three panels are raster plots of licking events during early (session 1), mid (session 4), and late (session 7) training stages. The bottom panel shows average licking rate over time (1-s bin) for each of the three sessions in the upper panels. Licks are aligned to the onset of CS1 (*t* = 0; the duration of CS1 (1 s) is indicated by a black bar above each panel). The delivery of US1 (water) was at 3 s after CS1 onset. Licks in the shaded area represent predictive licking events. **c** Eye blinking, measured as eye size change over time (see Methods), during the punishment learning for the same mouse as that in (**b**). The upper three panels are heat-maps of eye blinking during early (session 1), mid (session 4), and late (session 7) training stages. The bottom panel shows average blinking over time for each of the three sessions in the upper panels. Eye blinks are aligned to CS2 onset (*t* = 0; the duration of CS2 (1 s) is indicated by a black bar above each panel). The delivery of US2 (air-puffs) was at 3 s after CS2 onset. Eye blinks in the shaded area represent predictive blinking events. **d** and **e** The percentage of trials in which mice showed predictive licking (**d**) or blinking (**e**) (i.e., trials with at least one lick (**d**) or blink (**e**) event in the shaded area) in the first and last training sessions (the first 10 trials of each session were used for analysis) (*n* = 6 mice)

After imaging the activities of BLA neurons in naïve mice, we went on to train these mice in Pavlovian associative learning tasks and examined how these neurons might participate in learning. We trained the mice to first associate CS1 with US1 (reward learning), and then CS2 with US2 (punishment learning) (Fig. 2a; Methods). As the training progressed, the mice increased their licking in response to CS1 presentations (Fig. 2b, d; Supplementary Video 2) and eye closing (or “blinking”) in response to CS2 presentations (Fig. 2c, e; Supplementary Fig. 5, and Supplementary Video 3). These anticipatory responses provided measures of learning driven by US of either positive or negative valence. All mice reached high performance levels within seven sessions of training, licking the spout in anticipation of water delivery and blinking the eye in anticipation of air-puffing after CS onset and during the trace interval before US onset in over 90% of the trials (Fig. 2d, e).

**Fig. 3:**
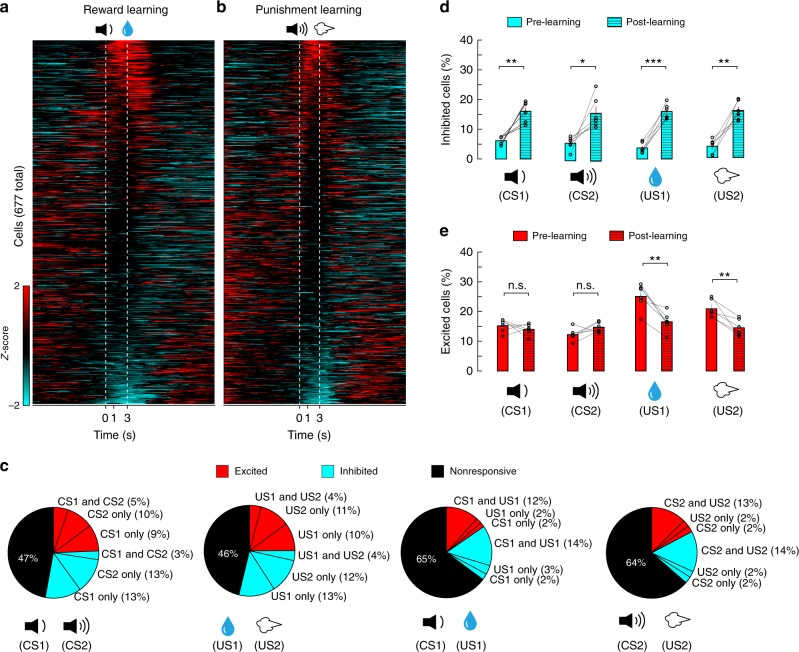
The CS and US responses in BLA neurons after learning. BLA neuronal activities were imaged in mice (*n* = 6) well trained with both the reward and the punishment conditioning. **a** Left: heatmaps of activities (*z*-scores) for all neurons (*n* = 677) in the reward block, in which CS1 was paired with US1 (indicated by the dashed lines). Each row represents the temporal activities of one neuron. Neurons are sorted according to their average *z*-scores during the 0–1 s time window. **b** Same as (**a**), except that imaging was performed in the punishment block, in which CS2 was paired with US2. **c** Pie charts showing the percent distributions of neurons responsive to different stimuli after learning. Note that at this stage, inhibitory responses became prominent; and the percentage of neurons responsive to both CS1 and US1, or to both CS2 and US2 increased. **d** Proportions of BLA neurons showing inhibitory responses to the CSs and USs before and after learning (*N* = 6 mice; CS1, *t*_(5)_ = −5.075, **P = 0.0039; CS2, *t*_(5)_ = −3.393, *P = 0.0194; US1, *t*_(5)_ = −9.413, ***P = 2.28e−4; US2, *t*_(5)_ = −5.886, **P = 0.002; paired *t*-test). **e** Proportions of BLA neurons showing excitatory responses to the CSs and USs before and after learning (CS1, *t*_(5)_ = 0.83, *P* = 0.44; CS2, *t*_(5)_ = −1.18, *P* = 0.29; US1, *t*_(5)_ = 5.24, **P = 0.003; US2, *t*_(5)_ = 4.194, **P = 0.008; paired *t*-test)

**Fig. 4:**
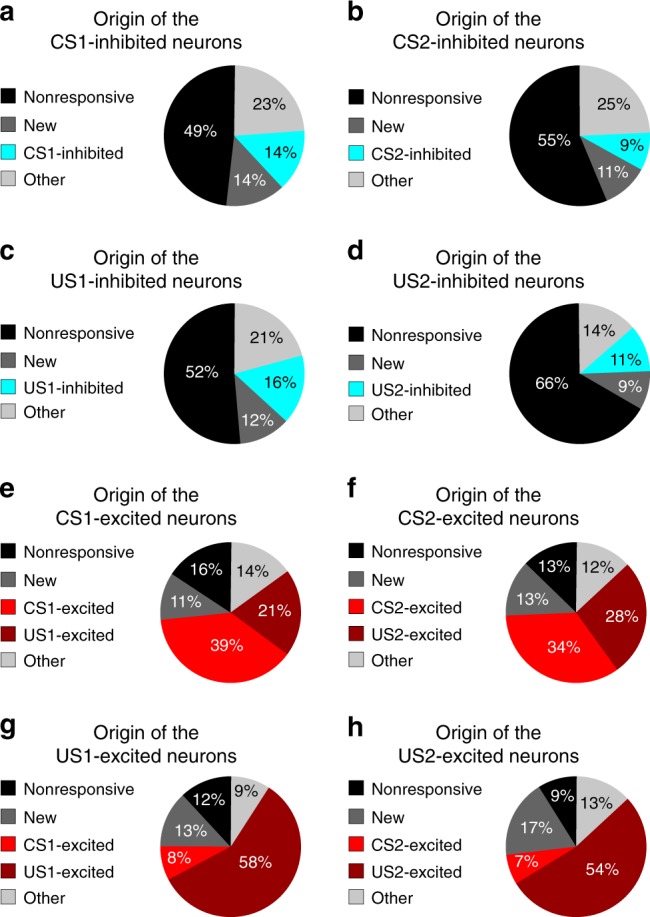
The origin of post-learning CS-responsive and US-responsive neurons. **a**–**d** For the BLA neurons showing inhibitory responses to CS1 (**a**), CS2 (**b**), US1 (**c**), or US2 (**d**) at the post-learning stage, the majority of them did not respond at the pre-learning stage to any of these stimuli (“nonresponsive”); some of them were not identified (“new”), were originally inhibited by CS1 (**a**), CS2 (**b**), US1 (**c**), or US2 (**d**) or were responsive to other stimuli (“other”) at the pre-learning stage. **e**, **f** For the BLA neurons showing excitatory responses to CS1 (**e**) or CS2 (**f**) at the post-learning stage, some of them did not respond at the pre-learning stage to any stimuli (“nonresponsive”); some of them were not identified (“new”), were originally excited by CS1 (“CS1-excited”) or US1 (“US1-excited”) (**e**) or by CS2 (“CS2-excited”) or US2 (“US2-excited”) (**f**), or were responsive to other stimuli (“other”) at the pre-learning stage. (**g**, **h**) For the BLA neurons showing excitatory responses to US1 (**g**) or US2 (**h**) at the post-learning stage, the majority of them were originally excited by US1 (“US1-excited”) (**g**) or US2 (“US2-excited”) (**h**) at the pre-learning stage; some of them did not respond to any stimuli (“nonresponsive”), were not identified (“new”), were originally excited by CS1 (“CS1-excited”) (**g**) or CS2 (“CS2-excited”) (**h**), or were responsive to other stimuli (“other”) at the pre-learning stage

**Fig. 5:**
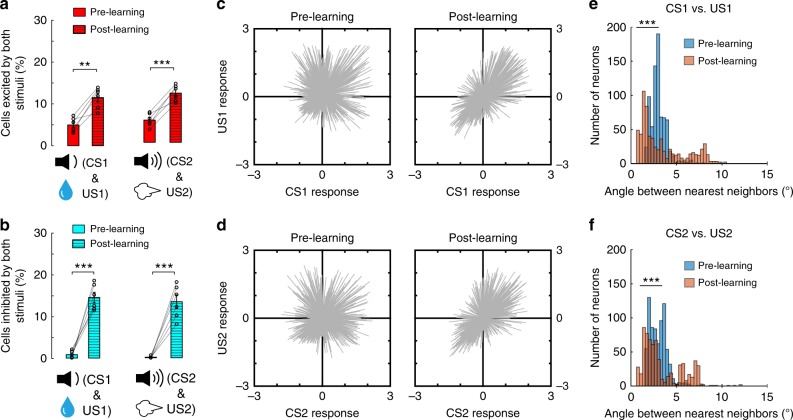
Learning links CS and US representations in the BLA. **a** The percentage of BLA neurons showing excitatory responses to both CS and US (*n* = 6 mice; CS1 & US1, *t*_(5)_ = −6.116, **P = 0.0017; CS2 and US2, *t*_(5)_ = −9.2, ***P = 2.5e−4; paired *t*-test). **b** The percentage of BLA neurons showing inhibitory responses to both CS and US (*n* = 6 mice; CS1 and US1, *t*_(5)_ = −8.8, ***P = 3.12e−4; CS2 and US2, *t*_(5)_ = −8.3, ***P = 4.11e−4; paired *t*-test). **c**, **d** The responses to CS1 and US1 (**c**), or to CS2 and US2 (**d**), for each neuron. Each line is a vector representing the responses of a particular neuron to both the CS and the US (values represent *z*-scores). Note that before learning, the vectors are distributed uniformly in the four quadrants, whereas after learning, the vectors are more concentrated in quadrants I and III. **e** The distribution of angles between the nearest neighbors among vectors in (**c**) (pre-learning median, 3.89, *n* = 756 neurons, post-learning median, 2.36, *n* = 677 neurons, *z* = 4.73, ***P = 2.26e−6, rank sum test). **f** The distribution of angles between the nearest neighbors among vectors in (**d**) (pre-learning median, 3.93, *n* = 756 neurons, post-learning median, 2.60, *n* = 677 neurons, *z* = 5.3, ***P = 1.42e−7, rank sum test)

In the last training session (equal to or earlier than the 7th session depending on the performance of individual mice) in the reward or punishment learning task, we imaged the activities of BLA neurons (*n* = 677) in these mice (*n* = 6) in response to CS1 followed by US1 (Fig. 3a) or CS2 followed by US2 (Fig. 3b), respectively. Compared with BLA neurons in naïve (pre-learning) mice, BLA neurons in the same but well trained (post-learning) mice showed interesting changes in their responses (Fig. 3c–e). First, the fractions of neurons showing inhibitory responses to the CSs or USs were all markedly increased (Figs. 1g and 3c, d). By contrast, the fraction of neurons excited by either CS1 or CS2 did not change (Fig. 3e), while that excited by either US1 or US2 was significantly reduced (Fig. 3e). The reduction in the number of US-excited neurons when the US was signaled by the CS is consistent with previous findings that expectation suppresses US responses of BLA neurons^23,48^. Interestingly, by tracking the same neurons before and after learning (Supplementary Fig. 6), we found that a major source of the neurons showing post-learning inhibitory responses were the nonresponsive neurons before learning (Fig. 4a–d). In contrast, the neurons showing post-learning excitatory responses to US1 or US2 were mainly those that were originally responsive to US1 or US2, respectively, before learning (Fig. 4g, h), whereas the neurons showing post-learning excitatory responses to CS1 or CS2 were derived from more divergent sources (Fig. 4e, f). Second, the fraction of neurons that were excited by both CS1 and US1, or by both CS2 and US2 (Fig. 5a; also see Figs. 1g and 3c), was more than doubled, despite the fact that the fraction of neurons excited by either US1 or US2 alone was significantly reduced (Fig. 3e). Third, the fraction of neurons that were inhibited by both CS1 and US1, or by both CS2 and US2 (Fig. 5b; also see Figs. 1g and  3c), was also markedly increased.

Overall, these changes suggest that learning substantially modifies the activity profile of BLA neurons. In particular, the second and third observations above (Fig. 5a, b) suggest that, after learning, a population of BLA neurons becomes to show consistent responses to both a CS and the associated US. Supporting this notion, further analysis revealed that the vectors representing the CS and US responses of individual neurons were distributed randomly before learning, but were mainly confined to two opposing quadrants after either the reward or punishment learning (Fig. 5c–f). Moreover, the CS and US responses in individual neurons were more correlated in the post-learning than pre-learning state (Supplementary Fig. 7). These results suggest that the CS is linked up with and thus becomes predictive of the ensuing US with learning in an ensemble of BLA neurons. This finding is consistent with previous studies showing that CS and US responses are correlated after learning at the single cell level^23,49^.

### Reward learning and punishment learning reduce noise correlations in the BLA

**Fig. 6:**
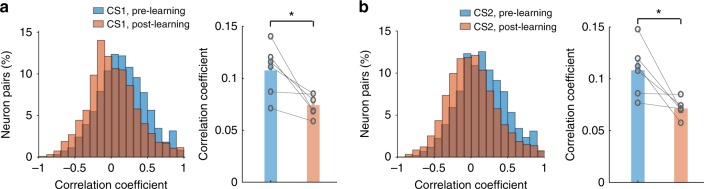
Learning reduces noise correlations in the BLA. **a** Left, histograms of noise correlations in the responses to CS1 in BLA neurons in a representative mouse. Right, comparison of noise correlations between pre-learning and post-learning conditions (*n* = 6 mice, *t*_(5)_ = 3.2, *P = 0.0241, paired *t*-test). **b** Left, histograms of noise correlations in the responses to CS2 in BLA neurons in a representative mouse. Right, comparison of noise correlations between pre-learning and post-learning conditions (*n* = 6 mice, *t*_(5)_ = 3.04, *P = 0.0288, paired *t*- test). The bar graphs on the right in **a** and **b** represent mean ± s.e.m

A critical feature of information encoding in neuronal ensembles is that noise correlations—the correlations between the responses of pairs of neurons to repeated presentations of an identical stimulus—affect the ability of downstream neurons to decode the information^50,51^. Cognitive processes such as learning and attention can reduce noise correlations in cortical areas^50–56^. To determine if such reduction occurs in the BLA, we computed the coefficients of noise correlations based on the CS responses of pairs of simultaneously recorded BLA neurons in each mouse (*n* = 6) (Methods), for both the pre-learning and the post-learning conditions (Fig. 6). The noise correlations during CS1 presentations and those during CS2 presentations were both reduced after learning (Fig. 6a, b). These reductions could be important for the expression of the valence-specific behavioral responses, as a reduction in noise correlations is thought to increase the signal-to-noise of population responses and behavioral performance^50–56^.

### Both reward and punishment transform CS representations in BLA neurons

**Fig. 7:**
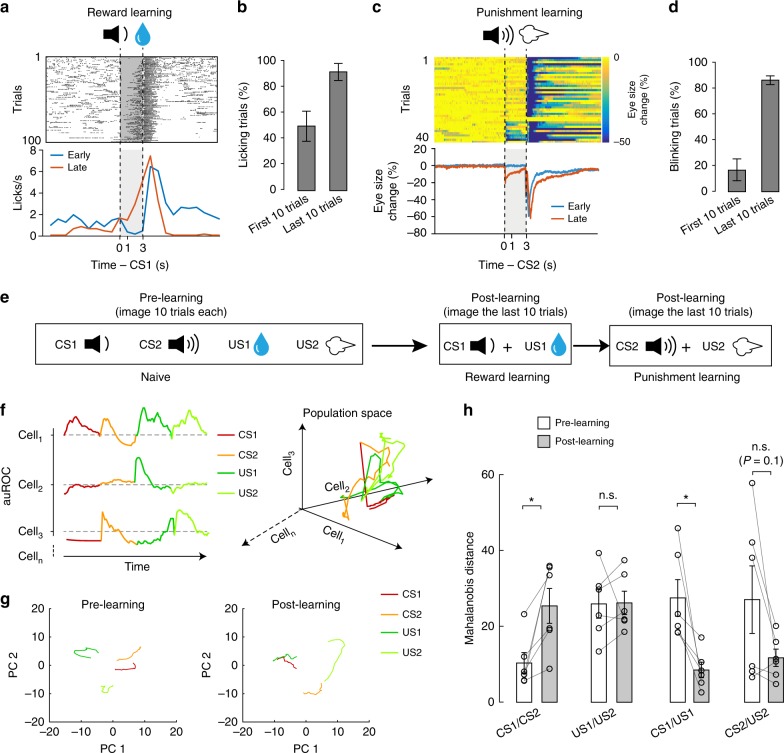
Learning transforms CS representations in the BLA. **a** Changes in licking during the reward learning for a representative mouse. Upper panel: raster plot of licking events across trials. Bottom panel: average licking rate over time (1-s bin), plotted separately for early and late trials. Dashed lines indicate the onsets of CS1 (1 s duration) and US1 (water). Licks in the shaded area represent predictive licking events. **b** The percentage of trials in which mice showed predictive licking in the first and last 10 trials (*n* = 3 mice). **c** Changes in eye blinking during the punishment learning for the same mouse as that in (**a**). Upper panel: heat-maps of eye size changes across trials. Bottom panel: average eye size changes over time, plotted separately for early and late trials. Dashed lines indicate the onsets of CS2 (1 s duration) and US2 (air-puff). Eye blinks in the shaded area represent predictive blinking events. **d** The percentage of trials in which mice showed predictive blinking in the first and last 10 trials (*n* = 3 mice). **e** The timing of the imaging experiments relative to behavioral training. **f** Left: a schematic of the population vector analysis. The dynamic activities of a neuron (cell_1_–cell_*n*_ for each mouse) during the 1 s time window immediately after the onset of each stimulus (CS1, CS2, US1, or US2) are represented by a vector, which is composed of sequential frame-by-frame *z*-scores computed for that neuron. Right: the trajectory of a population vector for three example neurons in a 3D space. **g** The BLA population activity from one representative mouse. The first two principal components before (left) and after (right) learning are projected onto a 2D space. **h** Quantification of the Mahalanobis distances between vectors representing neuronal responses to different stimuli (*n* = 6 mice; CS1/CS2, *t*_(5)_ = −3.25, *P = 0.022; US1/US2, *t*_(5)_ = −0.07, *P* = 0.94 (n.s., nonsignificant); CS1/US1, *t*_(5)_ = 3.29, *P = 0.021; CS2/US2, *t*_(5)_ = 2.01; *P* = 0.1; paired *t-*test). Data are presented as mean ± s.e.m

Our results thus far indicate that both reward learning and punishment learning profoundly change BLA neurons’ responses to the CSs, which are presumably important for guiding appropriate behaviors. However, it is unclear how these changes evolve during learning. To address this issue, we took advantage of the mice (3 out of 6) that learned both the reward and the punishment tasks within one session of training (Fig. 7a–d), and thus allowed us to track the activities of the same BLA neurons before and after learning without any change in imaging conditions (Fig. 7e) (see Methods).

As a first step to characterize how BLA neurons change their responses during learning, we classified these neurons (*n* = 518) into distinct functional types by hierarchical clustering following principal component analysis (PCA) on their responses to different stimuli, including CS1, CS2, US1 and US2 before and after learning in the reward and punishment learning tasks (Supplementary Fig. 8). This clustering yielded four types of neurons. Although there is heterogeneity within these types, the clustering captured some of their major features. On average, type I neurons (*n* = 130) reduced their activities in responses to CSs and USs after learning (Supplementary Fig. 8; Supplementary Fig. 9a–c); type II neurons (*n* = 187) showed stable or small reductions in their activities in responses to the CSs and USs after learning (Supplementary Fig. 8; Supplementary Fig. 9d–f); type III neurons (*n* = 128) increased and decreased their activities in response to CS1 and CS2, respectively, and increased their activities in response to both US1 and US2 after learning (Supplementary Fig. 8; Supplementary Fig. 9g–i); type IV neurons (*n* = 73) did not change their activities in response to CS1 and CS2, but reduced their activities in response to US1 after learning (Supplementary Fig. 8; Supplementary Fig. 9j–l). The more prevalent reduction in activities among BLA neurons after learning may, at least in part, account for some of the major effects of learning that we have observed, such as the marked increase in the fraction of neurons showing inhibitory responses to the CSs and USs (Figs. 1g, 3c, d and  4a–d).

To examine how the CS responses of BLA neurons as ensembles might transform during learning, we performed population analysis on the responses acquired from the same neurons before and after learning in individual mice (Fig. 7f–h) (*n* = 6 mice, of which three were the same as those used in Supplementary Figs. 8 and 9. For the other three mice that took several sessions to learn the tasks, neurons were tracked using the imaging registration methods (see Supplementary Fig. 6 and Methods)). Specifically, for each individual mouse, we used a vector to represent the dynamic activities of each neuron in response to the presentations of CS1, CS2, US1, and US2 (Fig. 7f), during both pre-learning and post-learning periods. Thus, each cell represented one dimension in an *n*-dimensional population space. We performed dimensionality reduction based on PCA, and used the reduced dimensions to represent the ensembles of responses (to CS1, CS2, US1 and US2) in each animal before and after learning (Fig. 7g). We then computed the Mahalanobis distance (MD) between ensemble representations as a measure of similarity (Methods) (Fig. 7h). Notably, we found that learning increased the MD between the ensemble representations of CS1 and CS2, but decreased that of CS1 and US1, and had a tendency to decrease that of CS2 and US2 (Fig. 7g, h). By contrast, learning did not change the distance between US1 and US2 (Fig. 7g, h). These results indicate that learning increased the discriminability of the representations of different CSs, but increased the similarity between the representation of a CS and that of a US. These results are consistent with the above observations that learning caused an increase in the fraction of neurons excited (Fig. 5a) or inhibited (Fig. 5b) by both CS1 and US1, or by both CS2 and US2.

### Reassignment of valences to CSs in the BLA during reversal learning

**Fig. 8:**
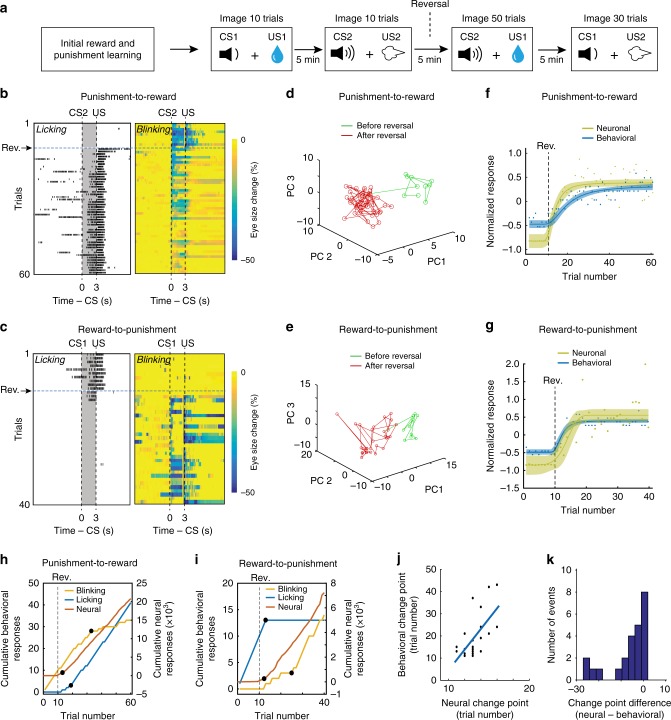
Population BLA activities correlate and predict behavioral responses during reversal learning. **a** A schematic showing the experimental procedure. The imaging procedure was designed such that no detachment/reattachment of the camera was needed, allowing ambiguous tracking of the same neurons throughout the reversal learning. **b** Punishment-to-reward reversal learning. Simultaneous measuring of licking (left) and blinking (right) behavior in the 10 trials before and 50 trials after the valence associated with CS2 changed from negative to positive. The horizontal dashed line denotes the first trial (11th trial) at which the valence was reversed. **c** Reward-to-punishment reversal learning. Simultaneous measuring of licking (left) and blinking (right) behavior in the 10 trials before and 30 trials after the valence associated with CS1 changed from positive to negative. The horizontal dashed line denotes the first trial (11th trial) at which the valence was reversed. **d**, **e** Projection of the trial-by-trial CS population responses (from 677 neurons) onto a 3D PCA space, in the punishment-to-reward (**d**) and the reward-to-punishment (**e**) reversal learning. **f**, **g** Average normalized neuronal and behavioral responses plotted as a function of trial number, for punishment-to-reward (**f**) and reward-to-punishment (**g**) reversal learning. **f** Fitting for behavioral responses, *r*^2^ = 0.86, fitting for neuronal responses, *r*^2^ = 0.83, Pearson correlation coefficient between behavioral and neuronal responses, *r* = 0.96, *P* < 0.001. **g** Fitting for behavioral responses, *r*^2^ = 0.91, fitting for neuronal responses, *r*^2^ = 0.74, Pearson correlation coefficient between behavioral and neuronal responses, *r* = 0.98, *P* < 0.001. Each of the dashed lines indicates the first trial (11th) at which the reversal of a CS valence has occurred. Shaded areas indicate 95% prediction intervals for Weibull fitting (*n* = 6 mice). **h**, **i** Cumulative plot of trial-by-trial measures of behavioral (licking and blinking) and neural responses of one representative animal in punishment-to-reward (**h**) and reward-to-punishment (**i**) reversal learning. Neural responses are represented as Mahalanobis distances between vectors representing the population CS response in a trial and a vector distribution that represents the population CS responses in all the 10 trials before the reversals (see text; also see **f**, **g**). Black dots represent the change points. **j** Correlation between behavioral change points and neural change points (*r*^2^ = 0.42, *P* = 6.3e−4, Pearson correlation). The blue line is the regression line. **k** Histogram showing the difference between neural and behavioral change points

To determine how BLA population responses to the CSs may be updated when CS–US contingencies change, and thus guide flexible behavior, we further trained the mice (*n* = 6) in a reversal learning procedure, in which the initial CS–US contingencies were switched (and thus the valences predicted by the CSs reversed) without warning (Fig. 8a). We simultaneously recorded behavioral (licking and blinking) and BLA neuronal responses in trials across the reversals (Fig. 8a–k). As expected, mouse behavior changed from anticipatory blinking to anticipatory licking following the punishment-to-reward reversal (Fig. 8b), and vice versa following the reward-to-punishment reversal (Fig. 8c). Because the mice reversed their behavioral responses quickly (within 50 and 30 trials, respectively, after the punishment-to-reward and reward-to-punishment reversal trials) (Fig. 8b, c), we were able to track the activities of the same population of neurons in each mouse in a single imaging session spanning all trials across the reversals (Fig. 8a–e).

To facilitate visualization of BLA population activities during the reversal learning, we projected the trial-by-trial population responses (*n* = 677 neurons) to presentations of the CSs onto a three-dimensional PCA space (Fig. 8d, e). For both the punishment-to-reward (Fig. 8d) and the reward-to-punishment (Fig. 8e) reversal learning, the trajectories of the responses before the reversal are clearly separable from those after the reversal, suggesting that the CS representations were reshaped or remapped onto different neural ensembles following the switch in CS–US contingencies.

Next, we examined the trial-by-trial relationship between BLA neuronal responses and behavioral reactions before and after the valence reversals (punishment-to-reward reversal, Fig. 8f; reward-to-punishment reversal, Fig. 8g). To quantify the changes in population activity in the BLA on a trial-by-trial basis in each mouse from pre-reversal conditions, we computed the MDs between vectors—each representing the population CS response in a trial—and a vector distribution, which represents the population CS responses in all the 10 trials before the reversals. These distances were normalized for each mouse and subsequently averaged for each trial (Fig. 8f, g). To quantify the changes in animal behavioral responses during the reversal learning, we normalized and combined the licking and blinking responses (see Methods). These behavioral responses were also averaged for each trial across animals. The trial-by-trial neuronal and behavioral responses were both fitted by sigmoidal Weibull functions, as previously described^24^ (Methods). We found that the changes in the trajectory of BLA population CS responses were highly correlated with those of the behavioral responses, in both the punishment-to-reward and the reward-to-punishment reversal learning (in both cases, Pearson correlation coefficients > 0.95, *P* < 0.001) (Fig. 8f, g). Furthermore, change point analysis^57^ (Fig. 8h, i) revealed that the changes in BLA neural activity were correlated with (Fig. 8j) and, in most cases, preceded (Fig. 8k) the changes in behavioral responses. These results suggest that the valence-specific expectation signals in the BLA at population level can be used to influence the on-going behavior.

Since the population CS responses of BLA neurons were strongly correlated with animals’ performance during reversal learning (Fig. 8f, g), we tested whether and when these neuronal responses can be used to reliably predict reward or punishment deliveries. We used the population responses to presentations of both the reward-predicting CS (CS-reward) and the punishment-predicting CS (CS-punishment) in the trials before, immediately after, or at the end of the valence reversals to train linear decoders to distinguish trials in which a CS-reward was presented from those in which a CS-punishment was presented (Supplementary Fig. 10a; Methods). We found that the decoder trained based on CS responses before or at the end of the reversals, when behavioral performance was high, had superior performance than the decoder trained based on CS responses immediately after the reversals (Supplementary Fig. 10b), a time point when behavioral performance was poor (Fig. 8b, c, f, g). These results suggest that the coding fidelity in BLA neural ensembles decreases immediately after a change in CS-US contingency but recovers with relearning.

Notably, only a fraction (25%) of neurons responsive to both CS1 and US1 (the water reward) before the reversal was responsive to both CS2 and US1 after the reversal when CS2 became to predict US1 (Supplementary Fig. 11). Similarly, a fraction (39%) of neurons responsive to both CS2 and US2 (the air-puff) before the reversal was responsive to both CS1 and US2 after the reversal when CS1 became to predict US2 (Supplementary Fig. 11). These results indicate that individual BLA neurons come in and out of memory ensembles during reversal learning.

## Discussion

In this study, we imaged BLA neuronal activities in mice across learning, including learning driven by both reward and punishment. Importantly, by using the imaging techniques we were able to track the activities of the same BLA neurons before and after learning, and during reversal learning in which the valences associated with the CSs were reversed unexpectedly. We found that the USs of opposing valences are represented by distinct but intermingled BLA neurons. Learning reduces noise correlations in the BLA and transforms CS representations in ensembles of BLA neurons, causing the population representations of CSs paired with reward and punishment to resemble those of the actual reward and punishment, respectively, and, as a result, causing the representations of CSs to be distinct from each other. This transformation is accompanied by the emergence of prominent inhibition and plasticity in the CS responses in individual BLA neurons. Furthermore, our results indicate that reversal learning induces remapping of CS representations onto BLA ensembles representing different valences, and that this remapping correlates and precedes the behavioral reversal, thus suggesting that BLA neuronal activities may be critical for the switch in behavioral actions following a change in CS–US contingency.

The prevalence of inhibition in BLA neurons induced by learning has not been noted by most previous studies. However, two recent studies^39,40^ have reported that a large fraction of BLA neurons is inhibited by the CS predicting either an appetitive or aversive US after conditioning. Interestingly, by tracking the same neurons across learning, we found that the BLA neurons showing post-learning inhibitory responses to the CSs and USs mainly evolve from the BLA neurons that are not responsive to any of these stimuli at the pre-learning stage (Fig. 4a–d; Supplementary Fig. 6). Inhibitory responses are more likely to be detected in BLA neurons displaying high basal firing rate, a property that has been described for a subset of GABAergic interneurons. It is therefore possible that such interneurons are part of the population showing inhibitory responses after learning. However, GABAergic neurons may not fully account for this population because, under our experimental conditions, only 5% of the BLA neurons expressing GCaMP6f were GABAergic (Supplementary Fig. 1b), whereas up to 30% of all the imaged neurons (and ~50% of all the responsive neurons) showed inhibitory responses after learning (Fig. 3c). Of note, recent studies have reported that identified BLA projection neurons can have a wide range of basal firing rate, from lower than 1 Hz to higher than 10 Hz^39,40,58^. In addition, as mentioned above, two of these studies^39,40^ showed that many BLA projection neurons are inhibited by CSs after conditioning, and that this population is as large as that excited by the same CSs—findings that are consistent with our results (Fig. 3c). We postulate that this inhibition mainly occurs onto BLA projection neurons and may result from the learning-dependent recruitment of inhibitory interneurons in the BLA, a process that has been shown to play an important role in learning during fear conditioning^31^. We further postulate that this inhibition may work in concert with the plasticity we observed (Supplementary Fig. 9) to shape the valence-specific CS representations during learning.

Our results are consistent with previous findings that different populations of neurons in the BLA respond to punishment and reward^22–24,35,37–40^, and that the valence-specific CS responses of BLA neurons at single cell level correlate with valence-specific behaviors in well-trained animals^23,24^. Our results are also consistent with recent findings in fear conditioning, in which learning drives the population representation of CS to match that of US in the BLA^43^. However, our study extends these previous studies by: (1) monitoring the activities of the same BLA neurons throughout the learning procedure—including pre-learning state and post-learning state—for both punishment learning and reward learning in the same animals, and thus revealing not only how BLA neurons differentially represent opposing valences but also how these representations evolve from naïve state through learning and (2) monitoring simultaneously neural activities and behavioral responses during reversal learning, to examine how changes in activities in BLA populations might occur and thus guide flexible behaviors. To our knowledge, our study is the first to address all these issues.

The main technique that we used in this study, the 1-photon (1P) imaging through GRIN lenses with miniscope, has lower spatial resolution than classical 2-photon (2P) imaging through GRIN lenses. However, the recently developed sophisticated imaging data extraction and analysis methods^47^ have mitigated this issue. In addition, the 1P-based method has the following advantages. First, it readily allows imaging simultaneously more than 100 cells in the field of view (FOV) under a GRIN lens of ~0.5 mm in diameter^43,59^ (and this study), as 1P imaging allows the collection of signals from cells outside of the focal plane. In comparison, much fewer cells can be obtained with 2P imaging in the FOV of a similar GRIN lens (e.g. refs. ^60,61^). Second, in imaging with a miniscope, the baseplate couples the camera to the GRIN lens and thus minimizes the drift in FOV across different imaging sessions, making it relatively easy to track the same neurons over many imaging sessions^62^ (Supplementary Fig. 6). Lastly, but not least, the setup for 1P imaging with miniscope is more affordable than a 2P setup to individual labs^46,63^.

The BLA is tightly connected to areas that are involved in the generation of behaviors motivated by negative or positive valence; it also receives inputs of all sensory modalities^11^. Thus, BLA neurons are anatomically poised to participate in the formation of CS–US associations and contribute to the establishment of behavioral responses driven by USs of different valences. Recent studies also provide evidence suggesting that some of the BLA neurons responsive to certain kinds of reward or punishment are hard wired and can be defined either genetically^35^ or by projection targets^37–40^. An important next step is to determine the relationship between the valence-specific BLA neurons classified on the basis of their in vivo activity^23,24^ (and the current study), expression of the immediately early genes or other genes^22,35,36^, and specific projection targets^37–40^.

## Methods

### Animals

Wild-type C57BL/6 mice (3 female, 3 male, 8–12 weeks old; The Jackson Laboratory) were used for all the experiments. Before surgery, mice were housed under a normal 12-h light/dark cycle (7 a.m. to 7 p.m. light) in groups of 2–5 animals, with food and water available ad libitum before behavioral training. After surgery, mice with GRIN lens implantation were housed singly. All behavioral experiments were performed during the light cycle. All animal procedures were approved and executed in accordance with Institutional Animal Care and Use Committees of Cold Spring Harbor Laboratory and with US National Institutes of Health standards.

### Viral vectors

The AAV1-Syn-GCaMP6f.WPRE.SV40 virus was purchased from the Penn Vector Core (Philadelphia, PA) and was used for expressing GCaMP6f in BLA neurons. The virus was stored in aliquots at –80 °C until use. We waited for at least 5 weeks after injection for sufficient viral expression.

### Stereotaxic surgery

Standard surgical procedures were used for stereotaxic injection and implantation, as previously described^34,64^. Briefly, mice were anaesthetized with isoflurane (3% at the beginning and 1% for the rest of the surgical procedure), and were positioned in a stereotaxic injection frame and on top of a heating pad maintained at 35 °C. A digital mouse brain atlas was linked to the injection frame to guide the identification and targeting of the amygdala (Angle Two Stereotaxic System, myNeuroLab.com). All subjects underwent two consecutive procedures in the same surgery: viral injection and GRIN lens implantation.

For each animal we made a small cranial window (1–2 mm^2^), through which a glass micropipette (tip diameter, ~5 μm) containing the GCaMP6 virus (1:4 or 1:8 dilution) was lowered down to the target. Virus (~0.3 μl) was delivered with pressure applications (5–20 psi, 5–20 ms at 0.5 Hz) controlled by a Picrospritzer III (General Valve) and a pulse generator (Agilent). The injection was performed using the following stereotaxic coordinates for the BLA: −1.6 mm from Bregma, 3.3 mm lateral from midline, and 4.5 mm vertical from cortical surface. The speed of injection was ~0.1 μl/10 min.

After virus injection, we waited for at least 10 min before removing the injection pipette. A GRIN lens (diameter, 0.6 mm; length, 6.7 mm; Inscopix) was then carefully implanted 200 μm above the center of the injection using a GRIN lens holder (Inscopix). The speed for lowering the GRIN lens was constant and slow (~100 μm/min). We secured the GRIN lens to the skull with C&B-Metabond Quick adhesive luting cement (Parkell Prod), and subsequently mounted a small piece of metal bar on the skull for head-fixation. Four to six weeks following GRIN lens implantation, we checked the fluorescent signals using a miniature microscope (nVista HD, Inscopix) in these mice under awake and head-fixation conditions. A baseplate (Inscopix) attached to the miniature microscope was then positioned above the GRIN lens. The focal plane was adjusted slowly until vascular structures and GCaMP6 dynamic activities were clearly observed^45^. The baseplate was subsequently secured with dental cement.

### Immunohistochemistry

Immunohistochemistry experiments were performed following standard procedures. Briefly, mice were anesthetized with Euthasol (0.2 ml; Virbac, Fort Worth, TX, USA) and transcardially perfused with 30 ml of PBS, followed by 30 ml of 4% paraformaldehyde in PBS. Brains were extracted and further fixed in 4% PFA overnight followed by cryoprotection in a 30% PBS-buffered sucrose solution for 36 h at 4 °C. Coronal sections (40 or 50 μm thickness) were cut using a freezing microtome (Leica SM 2010R, Leica). Sections were first washed in PBS (3 × 5 min), incubated in PBST (0.3% Triton X-100 in PBS) for 30 min at room temperature (RT) and then washed with PBS (3 × 5 min). Next, sections were blocked in 5% normal goat serum in PBST for 30 min at RT and then incubated with the primary antibody overnight at 4 °C. Sections were washed with PBS (5 × 15 min) and incubated with the fluorescent secondary antibody at RT for 2 h. After washing with PBS (5 × 15 min), sections were mounted onto slides with Fluoromount-G (eBioscience, San Diego, CA, USA). Images were taken using a LSM 780 laser-scanning confocal microscope (Carl Zeiss, Oberkochen, Germany). The primary antibody used was rabbit anti-GABA (Sigma, St. Louis, MO, USA; catalog number A2052). The fluorophore-conjugated secondary antibody used was Alexa Fluor^®^ 594 donkey anti-rabbit IgG (H+L) (Life Technologies, Carlsbad, CA, USA; catalog number A21207). Nuclei were stained with DAPI (4′,6-diamidino-2-phenylindole, Invitrogen, catalog number D1306; 0.5 µg/ml) for 15 min.

### Behavioral training

Water deprivation started 23 h before training in an auditory classical conditioning task, during which mice were head restrained using custom-made clamps and metal head-bars. Each mouse was first habituated to head restraint for 2–3 days prior to training. Unpredicted drops of water (5 µl) were delivered during the habituation. Once animals have learned how to lick, they were subjected to conditioning wherein two distinct auditory cues (conditioned stimuli, CS) were associated with different outcomes (unconditioned stimulus, US): a 2-kHz tone (1 s) predicted that a water reward (5 µl) was available from a metal spout next to the mouth, whereas a 10-kHz tone (1 s) predicted that an unpleasant air-puff (40 psi, 100 ms) would be blown to the face, in an area close to the eye. Animals were trained one session per day, with each session consisting of a reward block (100 trials) followed by a punishment block (30–50 trials). Each trial began with a CS, followed by a 2 s delay, and then a US. The inter-trial interval was randomly variable between 40 and 50 s.

Once mice have learned the initial associations (with the criterion that they correctly predicted the outcomes in more than 90% of the trials), they were further trained in a reversal learning session, in which we reversed the CS–US contingencies such that the CS initially associated with punishment became associated with reward (the punishment-to-reward reversal), and the CS initially associated with reward became associated with punishment (the reward-to-punishment reversal). Specifically, we first “reminded” mice with the original CS–US contingencies (10 trials of CS1-reward pairing, followed by 10 trials of CS2-punishment pairing). We then immediately subjected these mice to two blocks of reversal training, with each reversal being initiated without warning (50 trials of CS2-reward paring, followed by 30 trials of CS1-punishment pairing).

### Behavioral data collection and analysis

A custom software written in LabView (National Instruments) was used to control the delivery of CS and US and record behavioral responses, including licking and eye-blinking, during the CS-reward and CS-punishment conditioning. A metal spout was placed in front of the mouth of an animal for water delivery. The spout also served as part of a custom “lickometer” circuit, which registered a lick event each time a mouse completed the circuit by licking the spout. The lick events were recorded by a computer through the LabView software.

Eye blinking was tracked using a high-speed camera (FL3-U3-13S2C-CS, 120 HZ, Point Grey), which was controlled by a Bonsai software (Bonsai). Offline video analysis was conducted using EthoVision XT software (Noldus; Wageningen, The Netherlands). To measure the size of the eye, we manually selected a region of interest (ROI) surrounding the eye. Pixels corresponding to the eye were assigned as those that were darker than the surrounding background within the ROI. To quantify the changes in eye size (Δ*A*, which we refer to as “blinking”, although mice tend to close their eyes in response to an air-puff for a period much longer than that of a typical blinking event), we computed Δ*A*/*A*0(*t*) = (*A*(*t*) − *A*0)/*A*0, where *A*0 is the median size of the area corresponding to the eye during the 10-s baseline before CS onset and *A*(*t*) is the eye size in each picture frame, using a custom script written in MATLAB (The MathWorks, Inc., Natick, MA, USA). A blinking event was defined as Δ*A*/*A*0(*t*) < 20%.

### In vivo calcium imaging data acquisition and analysis

We followed a recently described procedure for the in vivo imaging experiments^34,45^. All imaging experiments were conducted on awake behaving mice under head-restraint in a dark, sound attenuated box. GCaMP6f fluorescence signals were acquired using a miniature integrated fluorescence microscope system (Inscopix, Palo Alto, CA) through GRIN lenses implanted in the BLA.

We installed a baseplate on top of the GRIN lens for each mouse, as described previously^34,45^. Before each imaging session, the miniature microscope was attached to the baseplate. The analog gain (1–3) and LED output power (10–40% of the maximum; 0.1–0.4 mW) of the microscope were set to be constant for the same subject across imaging sessions. The microscope was adjusted such that the best dynamic fluorescence signals were at the focal plane, which was subsequently kept constant across imaging sessions. To synchronize sensory stimuli and behavioral events with imaging acquisition, the Data Acquisition Box of the nVista Imaging System (Inscopix, Palo Alto, CA) was triggered by a behavioral control software written in LabView (National Instruments) through an NI data acquisition device (USB6008, National Instruments, CA). Compressed gray scale images were then recorded with nVistaHDV2 (Inscopix) at 10 frames per second. During imaging, the time stamps of different events, including the trigger signals sent to the microscope, CS, US, licks, and blinks, were all recorded with the behavioral control software (written in LabView) running on a dedicated high-speed computer.

We imaged BLA neuron activities during pre-learning, post-learning, and reversal learning sessions. To reliably detect stimulus-driven responses while minimizing photobleaching, we typically imaged neuronal responses to the same stimulus in 10 trials (except for the reversal learning; see below), with the imaging duration for each trial being 23 s to cover baseline, CS and/or US responses. During habituation, we imaged the responses to either CSs or USs, which were presented randomly interleaved.

During conditioning, three mice were able to learn both the CS-reward and the CS-punishment associations within the first session. We were thus able to image the same population of neurons in each of these mice before and after learning without the need of disassembling and reassembling the camera (Fig. 7). Thus, the imaging conditions (such as light power, focal plane, etc.) were kept constant throughout the learning process for these mice, allowing us to unambiguously track the same neurons or even compare the response amplitude before learning with that after learning for each neuron (Supplementary Fig. 9). For each of the other three mice, we carefully adjusted the camera to the similar focal plane as that in the pre-learning sessions, and then imaged the CS1–US1 and the CS2–US2 associations once the animal reached a high successful level (90%).

For reversal learning, we first acquired imaging data under the original CS–US contingencies (10 trials of CS1-reward pairing, followed by 10 trials of CS2-punishment pairing). This was immediately followed by imaging throughout the reversal learning (50 trials of CS2-reward paring, and then 30 trials of CS1-punishment pairing).

For imaging data processing and analysis, we first used Mosaic (version 1.0.0b; Inscopix, Palo Alto, CA) to combine all the video clips, each of which was recorded from one imaging session, into a single image stack (in TIFF format). The image stack was then spatially down sampled by a factor of 4 and corrected for motion artifacts using Mosaic. The motion-corrected video was next cropped to delete the margin areas.

Next, to address the problem of high levels of background fluorescence intrinsic to one-photon imaging, we applied the newly developed image analysis method, extended constrained non-negative matrix factorization (CNMF-E)^34,47^, which models the background with two realistic components: the constant baseline of each pixel, and the fluctuations from out-of-focus signals that is constrained to have low spatial-frequency structure. This decomposition avoids cellular signals being absorbed into the background term. After subtracting the background approximated with this model, we used CNMF to demix neural signals and get their denoised and deconvolved temporal activity, termed Δ*F*^65^. The CNMF-E method was carried out using a custom Matlab algorithm (for a detailed description of this method, see ref. ^47^). We then normalized Δ*F* by *F*0 to get Δ*F*/*F*0, where F0 is the modeled background fluorescence intensity.

To determine whether a neuron was significantly (*P* < 0.05) excited or inhibited by a stimulus, and thus can be classified as being “responsive” to the stimulus, we used the Wilcoxon signed-rank test to compare the mean Δ*F*/*F*0 values in the 1 s immediately after stimulus onset with those in the 1 s immediately before stimulus onset. For further analyses, such as the population analyses, we used *z*-scores to represent the dynamic activities in each neuron. To obtain the temporal *z*-scores for a neuron, we first obtained the mean activity trace for the neuron by averaging the fluorescence signals (Δ*F*/*F*0) at each time point across all trials, and then computed the *z*-scores as (*F*(*t*) – *Fm*)/SD, where *F*(*t*) is the Δ*F*/*F*0 value at time *t*, *Fm*, and SD are the mean and standard deviation, respectively, of the Δ*F*/*F*0 values over the entire peri-event period. For the trial-by-trial analyses, the *z*-scores were computed for each trial using the same method (but without the averaging across trials). We did not calculate the *z*-scores based on the mean and the standard deviation of Δ*F*/*F*0 values over a short baseline period, because in some cells such values can be zero, thus preventing meaningful *z*-score calculations (see also ref. ^66^).

### Cell registration

To identify the same individual cells from images acquired from different imaging sessions, we performed cell registration using a newly developed probabilistic method that automatically registers cells across multiple imaging sessions and estimates the registration confidence for each registered cell^62^ (Supplementary Fig. 6). Briefly, we first used the CNMF-E analysis to generate the spatial footprints for all cells imaged in the pre-learning session. We then repeated this process for the cells imaged in the post-learning session. We used the footprints from the pre-learning session as a reference map, and aligned with this map the footprints from the post-learning session by correcting for translation and rotation differences between different sessions. We subsequently calculated the probability of a given pair of cells, each from one of the two imaging sessions, to be the same cell (*P*_same_) based on their spatial correlation and centroid distance. A pair of cells is considered to have the same identity if *P*_same_ > 0.5. The centroid distance between a pair of cells deemed to have the same identity is generally small (≤6 µm).

### Classification of neurons with clustering analysis

Briefly, to classify neurons based on their CS and US responses before and after learning (Supplementary Fig. 8), we performed PCA on the *z*-scores representing the CS and US responses of these neurons^67^. We subsequently applied hierarchical clustering analysis to the first three principal components (PCs) using a correlation distance metric and complete agglomeration methods^64^.

### Analysis of noise correlations

Noise was defined as the trial-to-trial fluctuations around the mean in responses to repeated presentations of CS1 or CS2. Noise correlation was quantified as the Pearson correlation coefficient between such fluctuations in a pair of neurons. For this analysis, we used the mean *z*-score value in the 1-s time window immediately after CS onset to represent the CS response of a neuron in each trial.

### Population vector analysis

To investigate the learning-induced changes in the responses to CS or US at population level, we performed population vector analysis, adapted based on that described in a recent study^68^. Briefly, we created a series of *n*-dimensional (*n* equals the number of neurons) activity vectors by pooling the responses (*z*-scores) of individual neurons at each time point. Therefore, the ensemble BLA response at a particular time point is represented by a vector with a dimension equal to the total number of neurons in that ensemble. We used PCA for dimensionality reduction, and projected the population vectors onto a two-dimensional space for data visualization. To examine whether learning induced changes in BLA population responses to CS and US, we computed the MDs between vectors. For example, the MD between responses to CS1 and CS2 at each time point is defined by

$$\begin{array}{l}{\mathrm {MD}}\left( {\mathrm {{CS1,CS2}}} \right) = \\ \sqrt {\left( {{\mathrm {PV}}\left( {\mathrm {{CS1}}} \right) - {\mathrm {PV}}\left( {\mathrm {{CS2}}} \right)} \right)^T \ast {S}^{ - 1}\left( {\mathrm {{PV}}\left( {\mathrm {{CS1}}} \right) - {\mathrm {PV}}\left( {\mathrm {{CS2}}} \right)} \right)} \end{array}$$

where PV(CS1) and PV(CS2) are the population vectors of responses to CS1 and CS2, respectively. *S*^–1^ is the inverse of the covariance matrix.

### Correlation analysis of neural and behavioral responses

To compare the trajectory of changes in behavioral responses with that in neural responses, we averaged the normalized behavioral (licking and blinking) and neuronal responses for each animal in the 10 trials prior to and 50 (punishment-to-reward reversal) or 30 (reward-to-punishment reversal) trials after the valence reversal, as previously described^24^. In brief, we normalized the trial-by-trial behavioral responses by dividing them with the median response, followed by subtracting from the resulting responses the mean of the normalized values. To make the behavioral responses go from low to high, we multiplied the values by –1 for responses (licking or blinking) that were higher before than after the reversal. For neuronal responses, we first performed PCA on the trial-by-trial CS responses (*z*-scores) of all neurons in each mouse across reversal learning, and used the first three components to represent the population CS responses. We then computed the MD between the vector representing the population CS response in each trial and a vector distribution representing the population CS responses in all the 10 trials before the reversal. We normalized these distances, which represent the changes in CS responses during reversal learning, using a procedure similar to that used for normalization of the behavioral response. We next fitted both behavioral and neural data with sigmoidal Weibull functions:^24^

$$f\left( x \right) = u + \left( {1 - l} \right) \ast \exp \left( { - \left( {\frac{x}{\alpha }} \right)^\beta } \right)$$

where *x* is the behavioral or neural responses, *u* and *l* set the upper and lower asymptotes, respectively, and *α* and *β* determine the latency and abruptness, respectively, of the rise of the function. The Weibull functions model the averaged and normalized responses as a function of trial number.

To determine the relationship between neuronal responses and behavioral responses, we computed the Pearson correlation coefficients between the trial-by-trial neuronal responses and behavioral responses (licking or blinking) to the CS presentations.

### Decoding analysis

We performed the decoding analysis using the support vector machine (SVM) in MATLAB (MathWorks) to determine whether CS-reward and CS-punishment presentations could be predicted on the basis of BLA population CS responses acquired in each mouse at different stages of the reversal learning. We used *z*-scores to represent the trial-by-trial responses of each BLA neuron to presentations of either CS-reward or CS-punishment throughout the reversal learning. For each of the six mice, we captured the trial-by-trial population CS responses by a vector containing the responses of all neurons from that mouse, with each neuron representing one dimension in a multidimensional population space. We applied PCA on the multidimensional data from each mouse and used the first two PCs to represent the population response in each trial. We then used the low dimensional trial-by-trial population responses from each mouse to train and test binary linear classifiers with SVM to distinguish CS-reward presentations from CS-punishment presentations. Specifically, we used the responses from the 20 trials (10 CS-reward trials and 10 CS-punishment trials) immediately before, immediately after, or at the end of the valence reversals to train and test a classifier. For decoder training and testing, we used a 10-fold cross-validation procedure, in which all datasets were randomly partitioned to 10 equal-size subsamples, with each subsample containing equal number of responses from a given class (i.e., CS-reward vs. CS-punishment). We used 9 of the 10 subsamples for training and the remaining 1 for validating the decoder, and repeated this process 10 times such that each of the 10 subsamples was used exactly once as the validation data. The percentage of accurate classification incidence in the 10 times was reported as the final classification accuracy.

### Statistics and data presentation

All statistics are indicated where used. Statistic analyses were performed with Matlab (MathWorks, Natick, MA). All behavioral experiments were controlled by computer systems, and data were collected and analyzed in an automated and unbiased way. No statistical methods were used to pre-determine sample sizes. No randomization was used to assign experimental groups. No blinding was done. Virus-injected animals in which the injection site was incorrect were excluded. If the tract and tip of the GRIN lens was outside of the targeted area, the mouse was also excluded. No other mice or data points were excluded.

### Code availability

Matlab code used in this project for data analysis is available from the correspondence author upon reasonable request.

## Electronic supplementary material

Supplementary Information

Description of Additional Supplementary Files

Supplementary Movie 1

Supplementary Movie 2

Supplementary Movie 3

## Data Availability

The authors declare that the data supporting the findings of this study are available within the paper and its supplementary information files.
