# Supplementary material for: Population coding of valence in the basolateral amygdala
Source: Nat Commun. 2018 Dec 5;9:5195. doi: 10.1038/s41467-018-07679-9 (PMC6281657; doi:10.1038/s41467-018-07679-9)
Supplement: Supplementary file 1 — Supplementary Information [file 41467_2018_7679_MOESM1_ESM.pdf]

Population coding of valence in the basolateral amygdala

Zhang et al.

Supplementary Figures

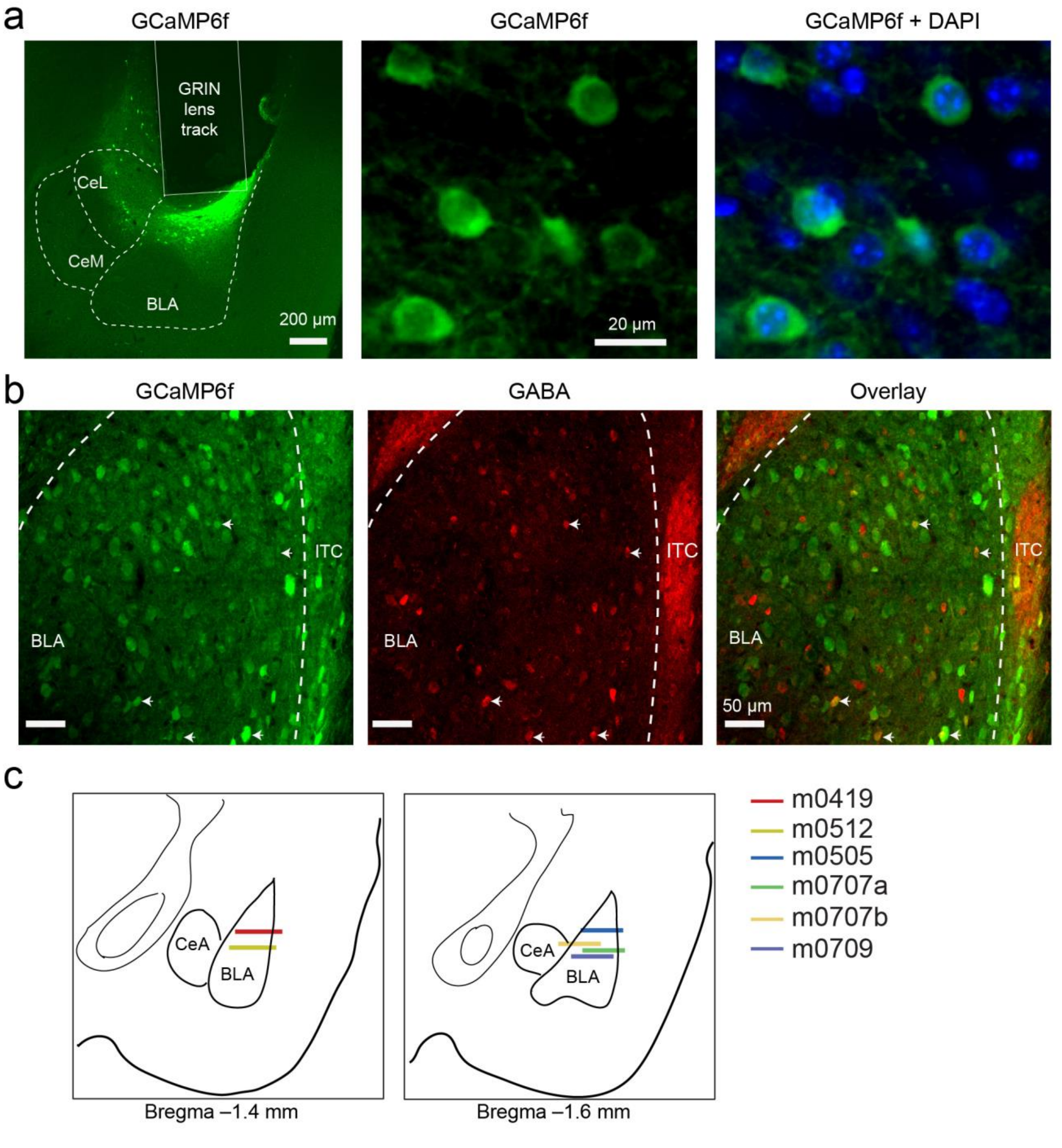

Supplementary Figure 1. Histology on virus infection and diagrams of GRIN lens implantation. (a) Left: an example low-resolution confocal image of the amygdala area, showing the track of an implanted GRIN lens and GCaMP6f expression in the BLA. Middle and right: high-resolution confocal images showing the GCaMP6f signals only (middle), and both the GCaMP6f signals (green) and the signals from DAPI (4′,6-diamidino-2-phenylindole) staining (blue) (right), in the BLA neurons. (b) The GCaMP6f-expressing BLA neurons were predominantly non-GABAergic. Shown are confocal images of the BLA infected with the AAV expressing GCaMP6f. GABAergic neurons were identified with an antibody recognizing GABA. White arrows indicate few cells that expressed both GCaMP6f and GABA (4.9±1% of GCaMP6f-expressing cells

expressed GABA; n = 3 mice). (c) Schematics showing the positioning of the tips of GRIN lenses implanted in the BLA of all the mice (n = 6) used in the imaging experiments.

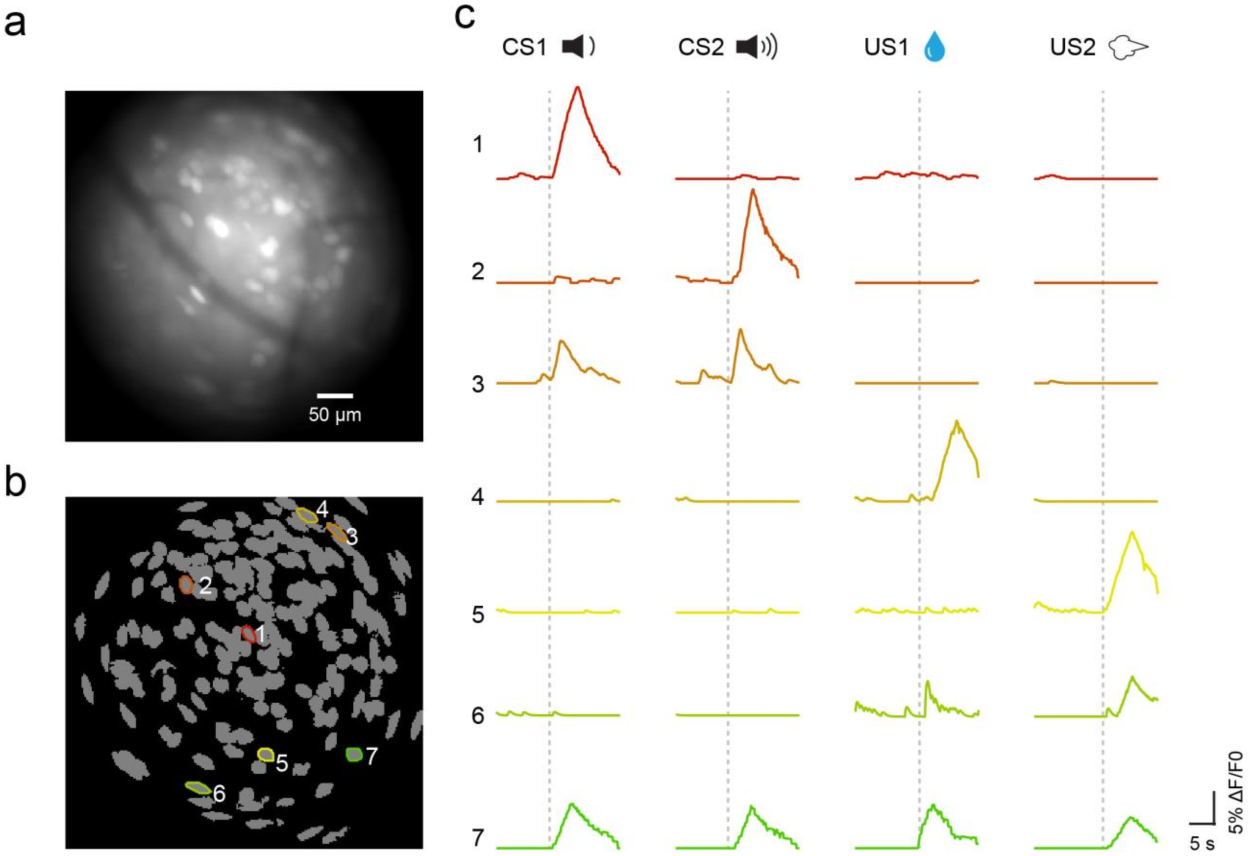

Supplementary Figure 2. Imaging BLA neuronal activities through GRIN lenses in behaving mice. (a) The field of view through a GRIN lens implanted in the BLA, showing the raw GCaMP6f fluorescence signals from BLA neurons acquired with the miniature microscope. (b) The spatial locations of individual extracted neurons (see Methods) in the field of view shown in (a). The contours of 7 representative neurons were outlined, color-coded and numbered. (c) The temporal calcium activities of the 7 neurons outlined in (b), color-coded and numbered in the same way. Each trace represents neuronal activities recorded from a single trial. Dashed lines indicate the onset of CS or US presentations.

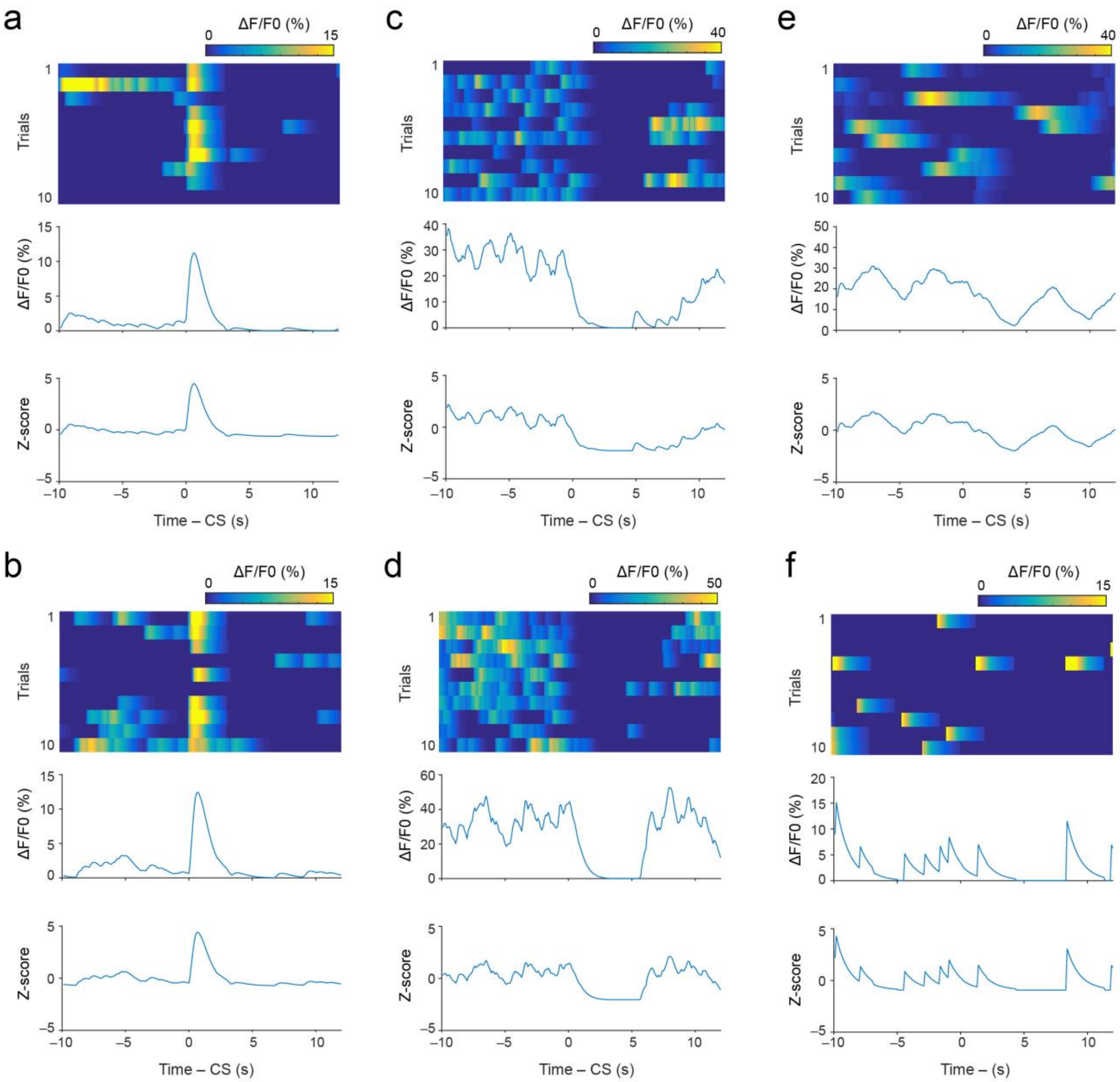

Supplementary Figure 3. Definition of stimulus-responsive neurons. In the top panels are heatmaps of trial-by-trial temporal calcium activities, represented as ΔF/F0 of GCaMP6f fluorescence signals, from six representative neurons that showed excitatory (a, b), inhibitory (c, d), or no (e, f) responses to CS2. The Dashed lines indicate the onsets of CS2 (t = 0). The Wilcoxon signed-rank test was used to compare, in all trials, the mean ΔF/F0 values in the 1s immediately before stimulus onset with those in the 1s immediately after stimulus onset. $P < 0.05$ is the criterion to define a stimulus-responsive neuron. Each of the middle panels is a ΔF/F0 plot generated by averaging the ΔF/F0 values at each time point across all trials for the neuron in the corresponding top panel. Each of the bottom panels is a z-score plot, calculated based on the ΔF/F0 values from the plot in the corresponding middle panel.

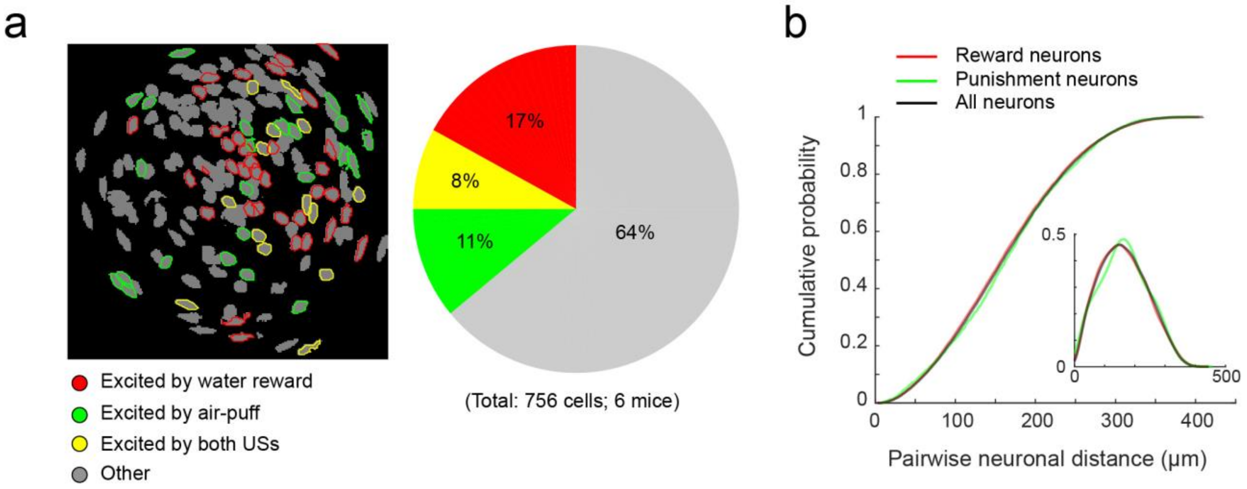

Supplementary Figure 4. BLA neurons responsive to appetitive or aversive stimulus are spatially intermingled. (a) Left: the spatial locations of individual extracted neurons (see Methods) in the field of view in the BLA of a representative mouse. The contours of neurons that showed significant excitatory responses to water reward, air-puff, or both stimuli were color-coded. The rest of the neurons ("Other") showed either inhibitory responses or no significant response to the two stimuli. The spatial distribution of these populations does not form obvious patterns. Right: pie graph showing the percentage distribution of these BLA populations (n = 756 cells from 6 mice). (b) Cumulative probability distributions of centroid distances between pairs of cells from the entire population ("all neurons"), between pairs of cells that were excited by the water reward ("reward neurons"), and between pairs of cells that were excited by the aversive air-puff ("punishment neurons") in each of the 6 mice (all neurons vs. reward neurons, P = 0.50; all neurons vs. punishment neurons, P = 0.08; K-S test). Inset, the corresponding probability densities.

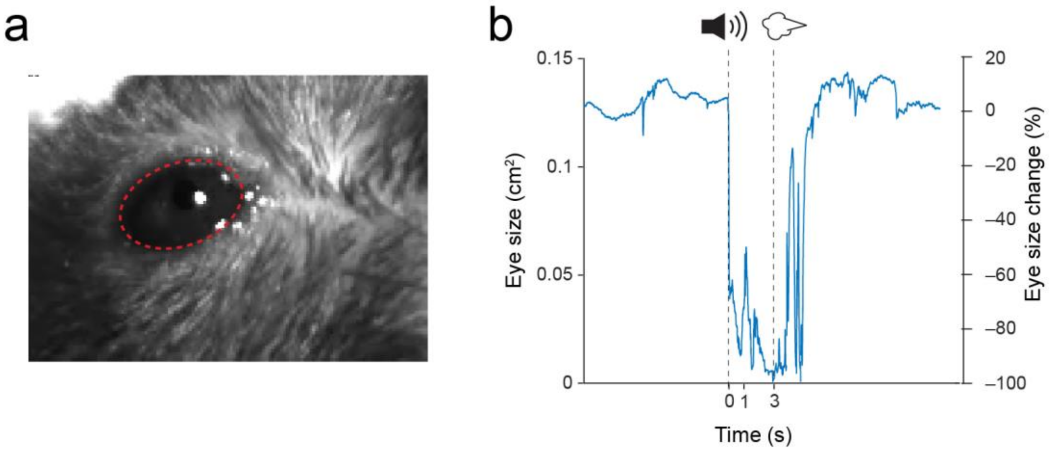

Supplementary Figure 5. Tracking the changes in eye size. (a) The eye of each mouse was tracked using a high-speed camera triggered by the behavioral control software, such that the tracking was synchronized with other events, including recording licking behavior or imaging neuronal activities. The size of the eye (outlined by the red circle) was measured offline. (b)

Tracking the eye size of a representative mouse during the punishment task. Dashed lines indicate the onsets of CS (1 s in duration) and US (air-puff). Both the absolute eye size (left y-axis) and the eye size change (right y-axis; see Methods) over time are indicated.

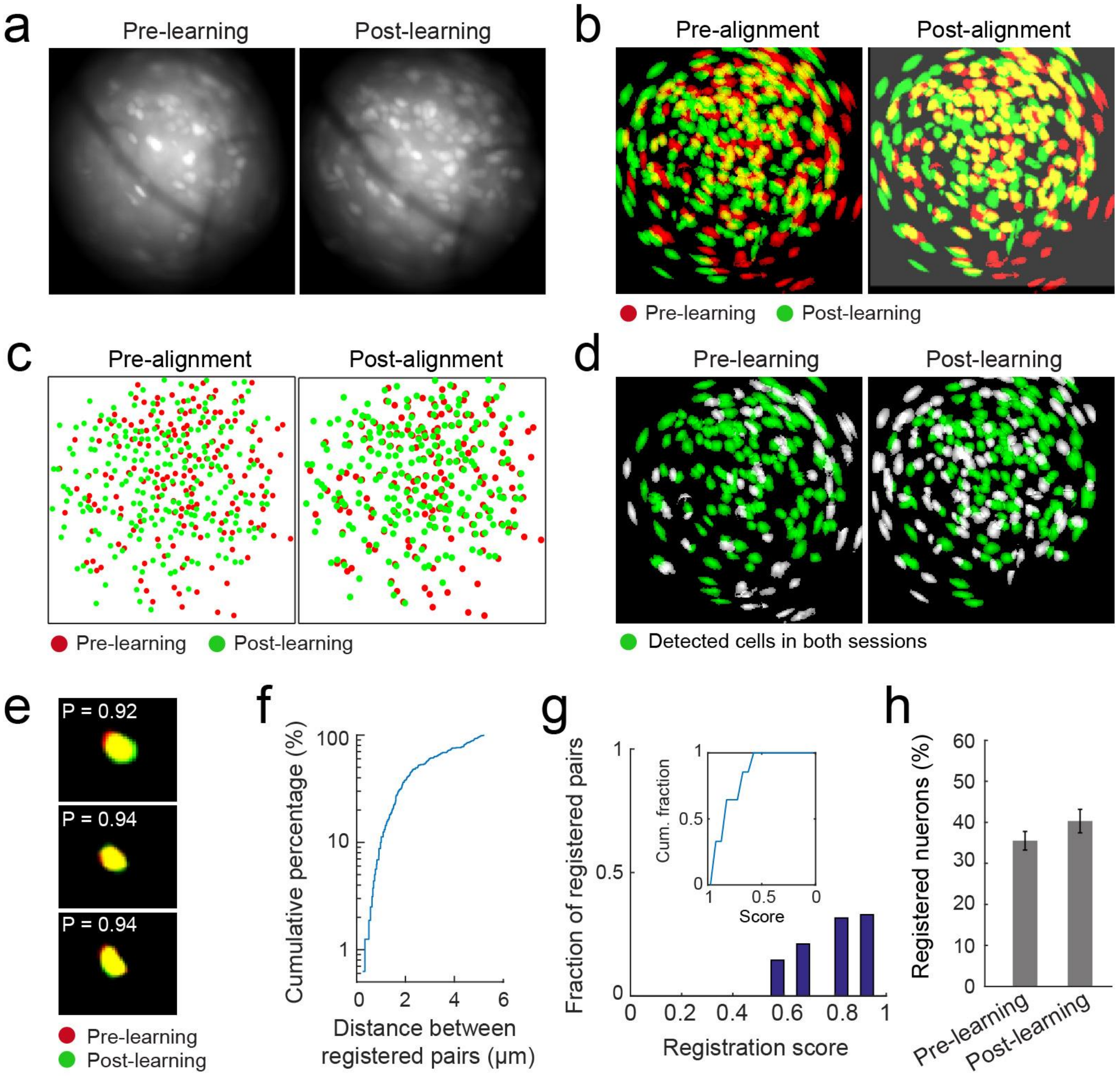

Supplementary Figure 6. Tracking the same neurons across learning. (a) Projections from the raw imaging data acquired in the pre-learning (left) and post-learning (right) sessions from a representative mouse. (b) Alignment of the imaging data from the pre-learning session with that from the post-learning session. The spatial footprints of all identified cells in the pre-learning (red) and post-learning (green) sessions are generated based on the CNMF-E analysis. (c) Same as in (b), except that a small dot was used to represent the centroid position of each neuron. (d) After the alignment, the neurons detected in both the pre-learning and the post-learning sessions are indicated in green. (e) Three representative pairs of neurons. Each pair contains two highly overlapping neurons – one identified in the pre-learning session (red) and the other in the post-learning session (green) – that are registered to be the same cell. The numbers indicate the spatial

correlations (P) for these pairs. (f) Cumulative distribution of distances between the centroids of all the registered pairs, like those in (e). (g) Distribution of registration scores for all the registered pairs. Inset, cumulative fraction of the registered pairs as a function of registration score from 1 to 0. (h) Percentage of registered cells in each of the two sessions.

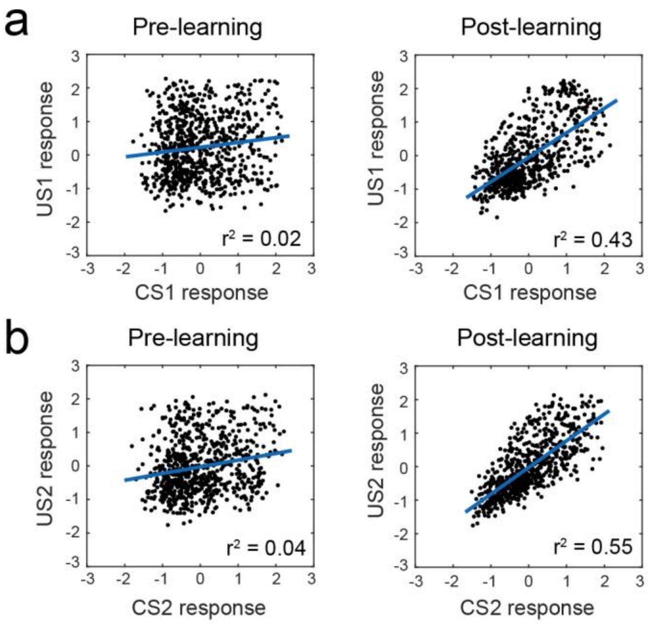

Supplementary Figure 7. Learning increases the correlation strength between CS and US responses in BLA neurons. The same data set as that in Fig. 5c, d was used for analysis. (a, b) The responses to CS1 and US1 (a), or to CS2 and US2 (b), for each neuron. Each dot represents the responses of a particular neuron to both the CS and the US (values represent z-scores). (a) Pre-learning, $r^2 = 0.02$, P = 0.0003, n = 756 neurons, post-learning, $r^2 = 0.43$, P = 1.09e–83, n = 677 neurons. (b) Pre-learning, $r^2 = 0.04$, P = 2.1e–8, n = 756 neurons, post-learning, $r^2 = 0.55$, P = 1.01e–118, n = 677 neurons. The blue line in each graph is the regression line for the corresponding data.

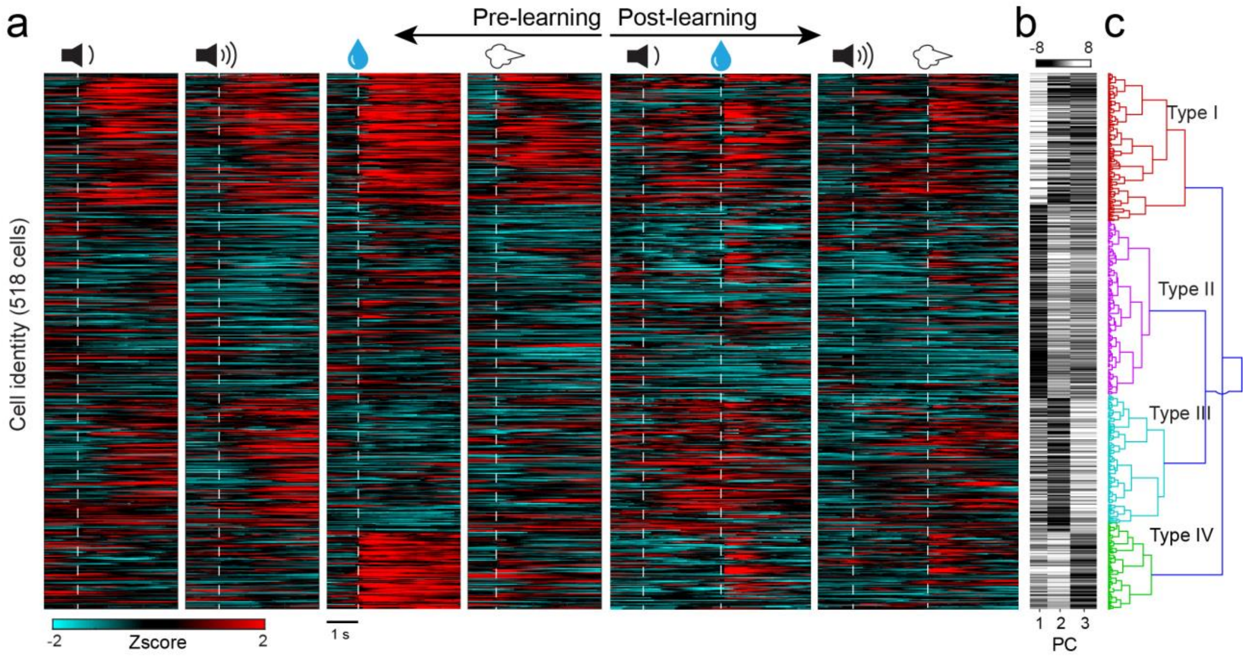

Supplementary Figure 8. Classification of BLA neurons based on their response profiles across learning. The same neurons were unambiguously tracked before and after learning.

(a) Heat-maps of the peri-stimulus activities (z-scores) of individual BLA neurons before and after learning. Each row represents the activities of one neuron (518 in total from 3 mice). Each of the dashed lines indicates the onset of a stimulus. (b) The first three principal components of the activity profile of each neuron. Each row represents the corresponding neuron in (a). (c) Hierarchy clustering of all neurons based on the PCA analysis.

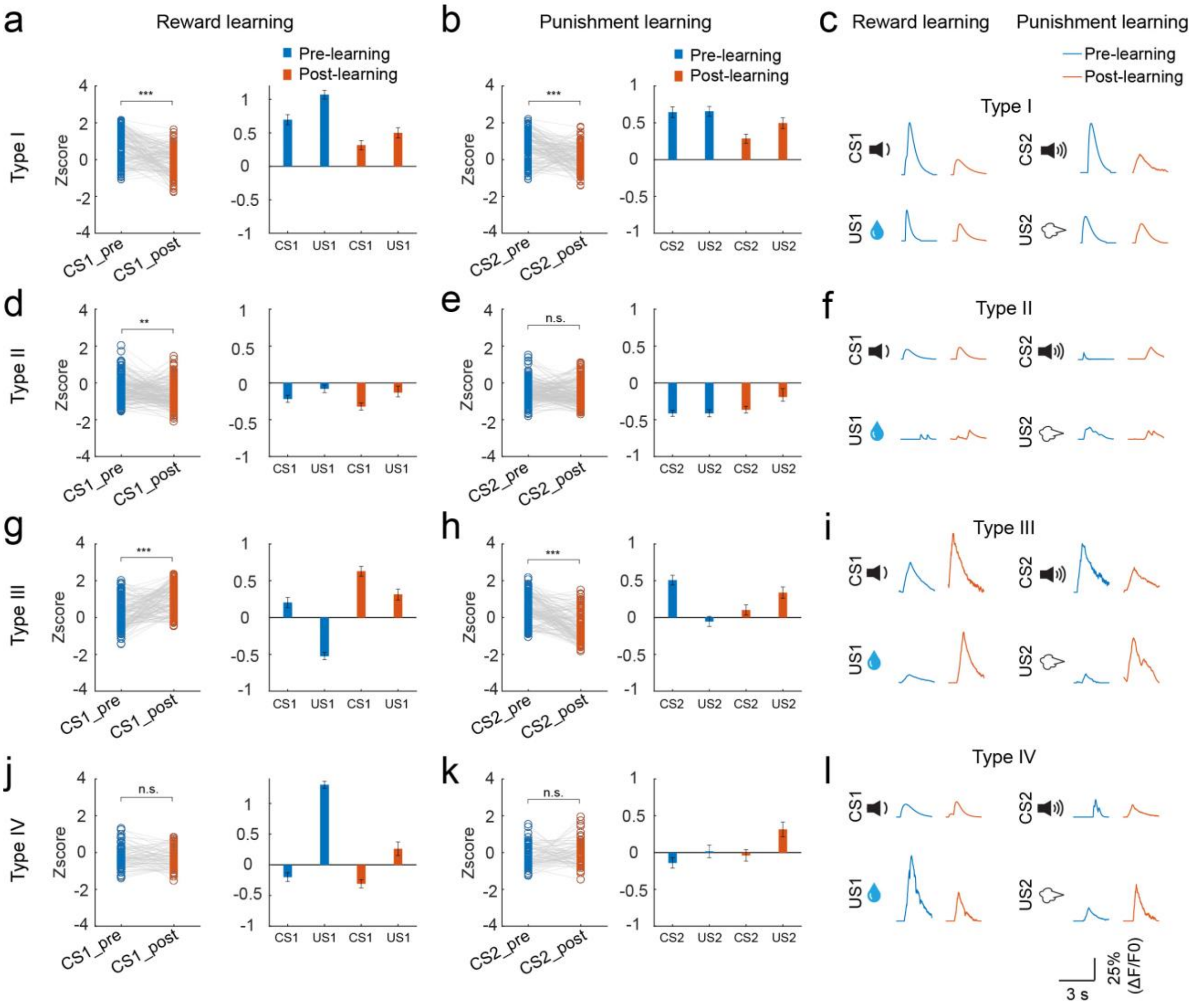

Supplementary Figure 9. Learning induced changes in functionally distinct classes of BLA neurons. For classification of neuronal types, see Supplementary Figure 8. (a-c) Learning induced changes in type I neurons. (a) Left: the responses of type I neurons to CS1 presentations before and after reward learning (n = 130 neurons, ***P = 7.2e–4, paired *t* test). Right: the responses of type I neurons to both CS1 and US1. The CS1 responses are the same as those shown in the left. (b) Left: the responses of type I neurons to CS2 presentations before and after punishment learning (n = 130 neurons, ***P = 1.0e–4, paired *t* test). Right: the responses of type I neurons to both CS2 and US2. The CS2 responses are the same as those shown in the left. (c) Responses of a type I neuron before and after reward learning, and those of a type I neuron before and after punishment learning. (d-f) Same as (a-c), except that the responses are from type II neurons (n = 187 neurons, **P = 0.008 in (d), P = 0.31 in (e), paired *t* test). (g-i) Same as (a-c), except that the responses are from type III neurons (n = 128 neurons, ***P = 2.4e–4 in (g), ***P = 4.7e–5 in (h), paired *t* test). (j-l) Same as (a-c), except that the responses are from type IV neurons (n = 73 neurons, P = 0.21 in (j), P = 0.36 in (k), paired *t* test).

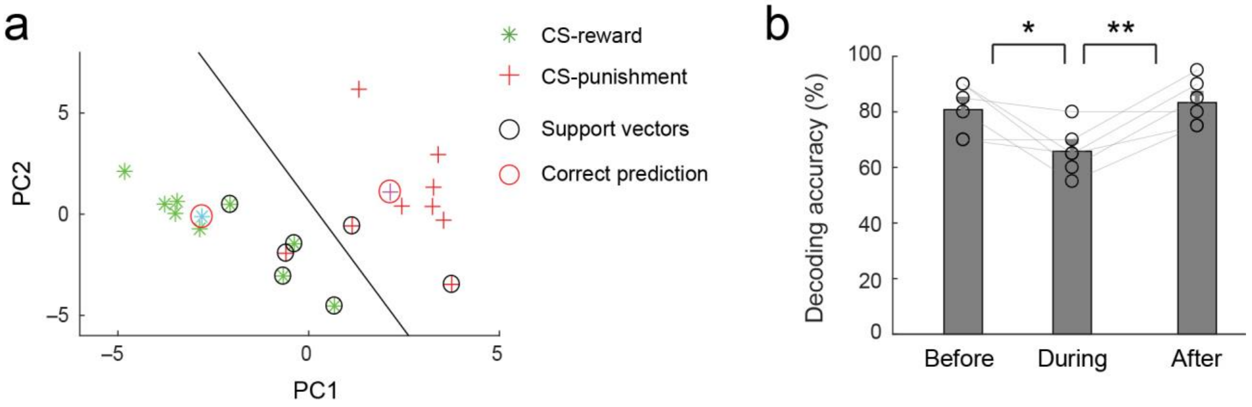

Supplementary Figure 10. Prediction of behavioral outcomes based on BLA population activities during reversal learning. (a) CS-reward and CS-punishment presentations are accurately classified by a linear decoder trained based on BLA population CS responses before the reversal in one mouse. (b) Performance of the decoders trained using population CS responses immediately before ("before"), immediately after ("during"), and at the end of ("after") the reversal ($n = 6$ mice, before vs. during, *$P = 0.037$, after vs. during, **$P = 0.009$, paired t test).

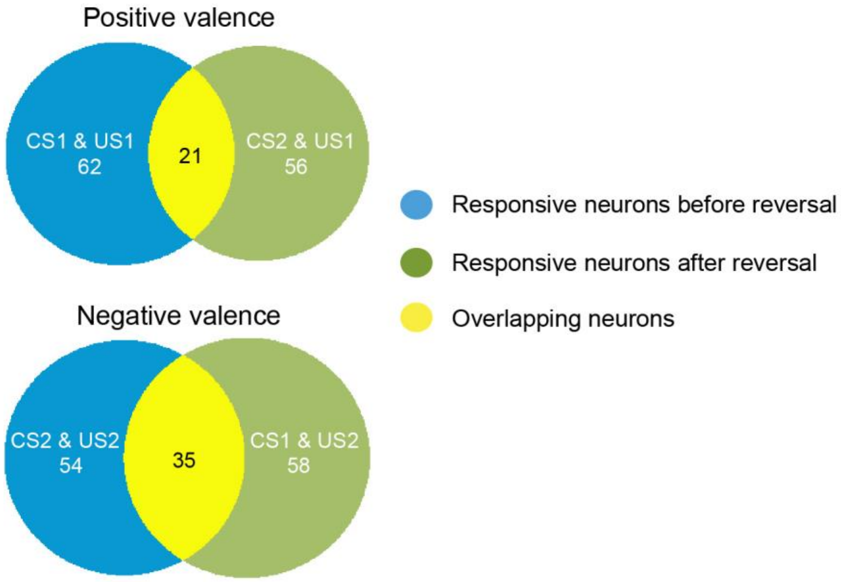

Supplementary Figure 11. Dynamic encoding of valence during reversal learning in the BLA. Upper panel: only a fraction (21/83) of neurons that was excited by both CS1 and US1 (the water reward) before the valence reversal was excited by both CS2 and US1 after the valence reversal, when CS2 became to predict US1. Lower panel: only a fraction (35/89) of neurons that was excited by both CS2 and US2 (the air-puff) before the reversal was excited by both CS1 and US2 after the reversal, when CS1 became to predict US2.
