# Supplementary material for: Population coding of valence in the basolateral amygdala
Source: Nat Commun. 2018 Dec 5;9:5195. doi: 10.1038/s41467-018-07679-9 (PMC6281657; doi:10.1038/s41467-018-07679-9)
Supplement: Supplementary file 2 — Description of Additional Supplementary Files [file 41467_2018_7679_MOESM2_ESM.pdf]

Description of Additional Supplementary Files

File Name: Supplementary Movie 1
Description: **Extraction of the spatial and temporal components of neuronal activity from GCaMP6 signals in BLA neurons imaged through GRIN lenses.** Shown are videos of GCaMP6f signals from BLA neurons in a representative mouse receiving air-puffs. These videos are short clips of movies stitched together, with each clip representing a trial (the onset of which is denoted by each of the solid black lines in the bottom right panel). These videos are played in synchrony, and represent the following contents: "**raw data**", GCaMP6 signals without any processing; "**background**", the background components in the raw data approximated with our model; "**raw–background**", the remaining signals after subtraction of the background from the raw data; "**residual**", the remaining signals; "**denoised**", the denoised and deconvolved spatiotemporal activity for each neuron obtained from the "raw–background" signals using the CNMF-E (Methods). In the "denoised" video, the contours of 3 representative neurons are traced and numbered. The temporal activities of these 3 neurons are displayed in the bottom right panel, in which the moving line indicates the passage of time synchronized with all the videos; the stationary solid black lines denote the onsets of trials (and thus the junctions between the short movie clips); and the dashed red lines denote the onsets of air-puffs. Neurons #1 & 2 are air-puff-responsive neurons, whereas neurons #3 only shows spontaneous activities. The scale bar beside each of the videos denotes ΔF values. See Methods for a more detailed description.

File Name: Supplementary Movie 2
Description: **Licking behavior driven by the reward cue.** This video shows the licking behavior of a thirsty mouse after presentation of a sound (delivered at 10 s) predicting water reward (delivered at 13 s).

File Name: Supplementary Movie 3
Description: **Blinking behavior driven by the punishment cue.** This video shows the blinking behavior of a mouse after presentation of a sound (delivered at 10 s) predicting air-puff (delivered at 13 s).
